# Rhodopsin 7–The unusual Rhodopsin in *Drosophila*

**DOI:** 10.7717/peerj.2427

**Published:** 2016-09-06

**Authors:** Pingkalai R. Senthilan, Charlotte Helfrich-Förster

**Affiliations:** Neurobiology and Genetics, Biocenter, University of Würzburg, Würzburg, Germany

**Keywords:** Rhodopsins, Opsins, GPCR, *Drosophila*, Vision, Phototransduction

## Abstract

Rhodopsins are the major photopigments in the fruit fly *Drosophila melanogaster. Drosophila* express six well-characterized Rhodopsins (Rh1–Rh6) with distinct absorption maxima and expression pattern. In 2000, when the *Drosophila* genome was published, a novel *Rhodopsin* gene was discovered: *Rhodopsin 7* (*Rh7*). *Rh7* is highly conserved among the *Drosophila* genus and is also found in other arthropods. Phylogenetic trees based on protein sequences suggest that the seven *Drosophila* Rhodopsins cluster in three different groups. While Rh1, Rh2 and Rh6 form a “vertebrate-melanopsin-type”–cluster, and Rh3, Rh4 and Rh5 form an “insect-type”-Rhodopsin cluster, Rh7 seem to form its own cluster. Although Rh7 has nearly all important features of a functional Rhodopsin, it differs from other Rhodopsins in its genomic and structural properties, suggesting it might have an overall different role than other known Rhodopsins.

## Introduction

Higher animals are able to perceive their environment through various senses, and vision is possibly the most crucial one. Not only is it well-developed in many animals but it is also the most sensitive sense. A single photon is sufficient to activate a signaling cascade in which many different phototransduction proteins are involved (Nikolic et al., 2010). Rhodopsins, photon-receiving G-protein coupled proteins, play a decisive role in this context. Rhodopsins are seven helix membrane proteins that are covalently coupled to a light sensitive chromophore, called retinal. Photon absorption leads to the isomerization of retinal, which results in the conformational change of Rhodopsin to Metarhodopsin and subsequent activation of the G-protein coupled cascade (Devary et al., 1987; Britt et al., 1993; Kiselev & Subramaniam, 1994).

**Figure 1:**
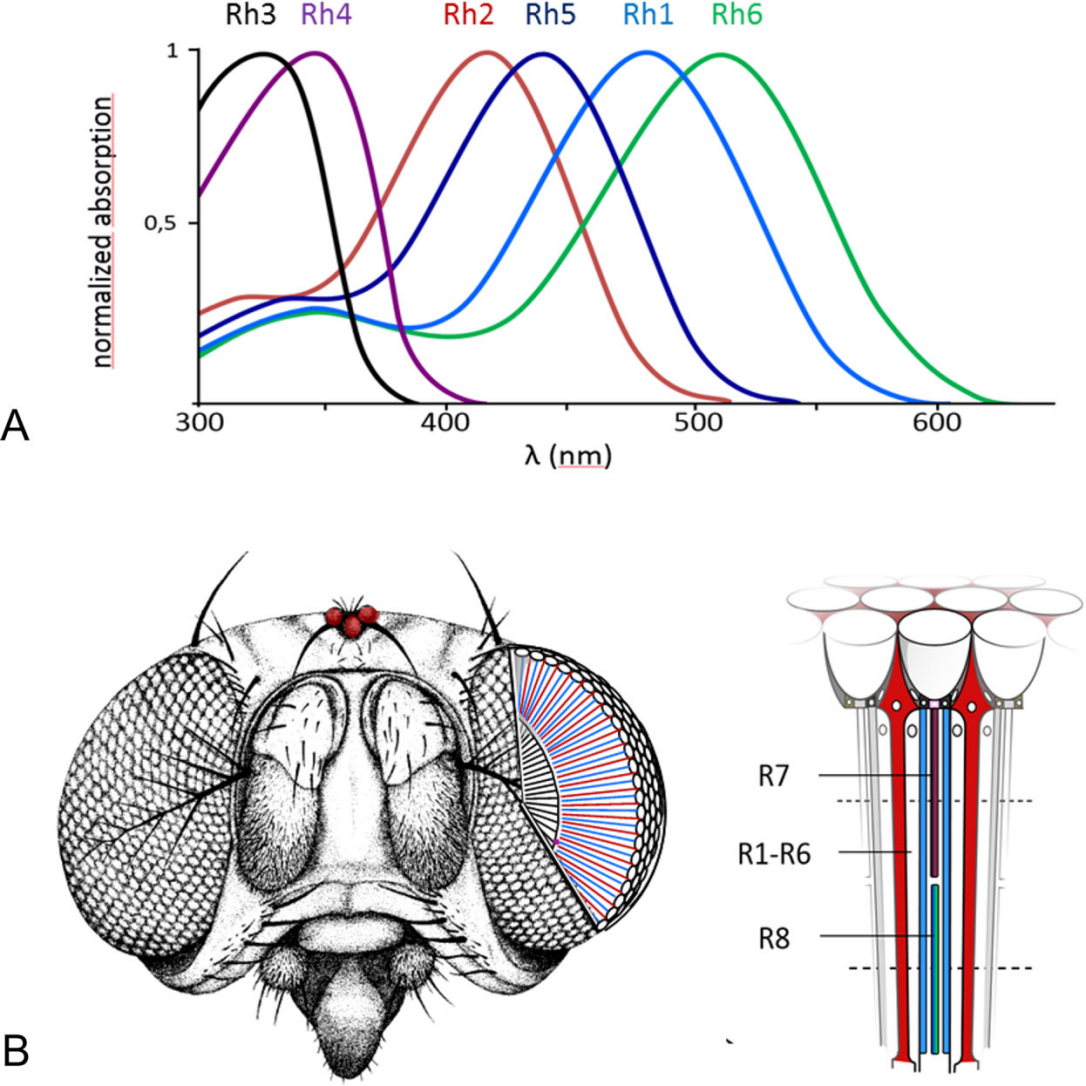
Six known *Drosophila* Rhodopsins. (A) All known Rhodopsins have a distinct absorption maxima ranging from UV light (331 nm) to visible green light (515 nm) (adapted from Stavenga & Arikawa (2008)). (B) While Rh2 is expressed in fly’s ocelli, all other known Rhodopsins are primarily expressed in different receptor cells of the compound eye (modified from Helfrich-Förster (2014) and Grebler (2015)).

To have a broad and sensitive visual system, many animals express more than one Rhodopsin molecule. The fruit fly *Drosophila melanogaster* possesses six well-characterized Rhodopsins with distinct spectral sensitivities, named Rh1–Rh6 (Fig. 1A). While Rh1, Rh2 and Rh5 are sensitive to blue light (absorption maxima of 486, 418 and 442 nm), Rh3 and Rh4 are sensitive to UV light (331 and 355 nm), and Rh6 is sensitive to green light (515 nm) (Salcedo et al., 1999).

Besides differences in spectral sensitivity, each Rhodopsin also exhibits differences in expression site (Chou et al., 1999) (Fig. 1B). While Rh2 is expressed in the dorsal ocelli, the simple primitive eye found in many invertebrates, all other Rhodopsins (Rh1, Rh3, Rh4, Rh5, and Rh6) are expressed in different photoreceptor cells of the compound eye. The compound eye consists of approximately 800 identical optical units, termed ommatidia, and each ommatidium contains eight photoreceptor cells (R1–R8). Each photoreceptor cell expresses only one type of Rhodopsin: the outer photoreceptor cells (R1–R6) express the major Rhodopsin Rh1, which is encoded by the gene *ninaE* (O’Tousa et al., 1985; Zuker, Cowman & Rubin, 1985), the distal inner receptor cell R7 expresses either Rh3 or Rh4 whereas the proximal inner receptor cell R8 express either Rh5 or Rh6 (Rister, Desplan & Vasiliauskas, 2013; Behnia & Desplan, 2015). Two types of ommatidia, pale and yellow, are randomly interspersed in the compound eye, i.e. pale ca. 30% and yellow ca. 70%. The pale-type ommatidia contain Rh3 in the distal inner cell R7 cell and Rh5 in the proximal inner cell R8, while the yellow-type ommatidia contain Rh4 in the distal inner cell R7 and Rh6 in the proximal inner cell R8 (Chou et al., 1996; Huber et al., 1997; Papatsenko, Sheng & Desplan, 1997).

However, Rhodopsin expression seems not to be restricted to the eyes and to the ocelli. Two Rhodopsins, Rh5 and Rh6, are also found in the Bolwig’s organ in the larvae (Yasuyama & Meinertzhagen, 1999; Sprecher & Desplan, 2008) and in the H.B-eyelet (Hofbauer & Buchner, 1989; Helfrich-Förster et al., 2001) of the adult fly. Though not as much as in in visual organs, Rhodopsins are also found in non-visual organs, e.g. in the testis (Alvarez, Robison & Gilbert, 1996), in the body wall (Shen et al., 2011), and in the second antennal segment (Senthilan et al., 2012). This might indicate that Rhodopsins are more than only visual proteins as they appear to be involved in audition and thermosensation. In addition, Rhodopsins are important for synchronizing the fly circadian clock to the environmental light-dark-cycles (Hanai & Ishida, 2009; Rieger, Stanewsky & Helfrich-Förster, 2003; Schlichting et al., 2014). In mammals, a special Rhodopsin, Melanopsin, that has all the characteristics of an invertebrate Rhodopsin, has a prominent role in synchronizing the circadian clock (reviewed in Lucas, 2013). In the fly, Melanopsin is not present but the existence of a further Rhodopsin was predicted from a study showing that flies that lack all Rhodopsins plus the blue-light photopigment Cryptochrome still respond to light by decreasing activity in the morning, even though the presence of this Rhodopsin alone was not sufficient to entrain the endogenous clock to the light-dark cycles (Helfrich-Förster et al., 2001).

Indeed, when the genome of the fruit fly *Drosophila melanogaster* was published in the year 2000, a novel Rhodopsin gene appeared. This gene *CG5638* was then called Rhodopsin 7 (Rh7) due to the sequence similarity to other Rhodopsin genes. In this study, we tested the structural and evolutionary characteristics of Rh7 and compared it with other known Rhodopsins to get a first idea about its putative function. Not to be biased by putative pseudogenes, we compared the sequences of the Rhodopsin proteins and not those of the genes.

## Materials and Methods

### Protein sequences

All *Drosophila* Rhodopsin sequences were obtained from Flybase (http://www.flybase.org; FB2016_02, released March 30, 2016). To calculate the E-values, the Flybase BLAST (http://flybase.org/blast/; blastp 2.2.18 Mar-02-2008) was used (Altschul et al., 1997).

Based on annotated protein databases and the blastp program in NCBI (http://blast.ncbi.nlm.nih.gov/Blast.cgi), we identified putative homologues for the *Drosophila* Rhodopsins and for other opsins. Putative homologues, especially from Rh7, were re-blasted in http://flybase.org/blast/ to confirm that they are indeed homologues of the *Drosophila* Rhodopsins. All Protein sequences were then exported and characterized in GENtle V.1.9.4. (http://gentle.magnusmanske.de).

### Sequence identities and similarities

Sequence identities and similarities were calculated with Sequence Identities and Similarities (SIAS) (SIAS, April, 2008; http://imed.med.ucm.es/Tools/sias.html). For the similarity calculation, the default parameters offered by SIAS were chosen, in which all positively charged amino acids (Arg, Lys, and His), all negatively charged amino acids (Asp and Glu), and all aliphatic amino acids (Val, Iso, and Leu) were considered as similar. Additionally the aromatic amino acids Phe, Tyr, and Trp, the polar amino acids Asn and Gln, and the small amino acids Ala, Thr, and Ser were treated as similar. To calculate the normalized similarity score, the BLOSUM62 matrix was chosen. Protein sequences were aligned with the Clustal-W algorithm in the GENtle software.

### Phylogeny analyses

Multiple alignments were then exported to Phylogeny.fr (http://www.phylogeny.fr/). Phylogenic tree analyses were then performed using the One Click method. In this method the MUSCLE 3.8.31 multiple alignment, the Gblocks 0.91 b Alignment refinement, the PhyML 3.1/3.0 aLRT Phylogeny (using the maximum likelihood), and the TreeDyn 198.3 tree rendering are used by default (Dereeper et al., 2008; Dereeper et al., 2010).

### Further in silico analysis

The hydrophobicity analyses were performed with PolyPhobius (http://phobius.sbc.su.se/poly.html), a combined transmembrane topology and signal peptide predictor (Käll, Krogh & Sonnhammer, 2005). To find out whether Rhodopsin Core Sequence I (RCSI) is located in the genome of different *Drosophila* species, the sequence of the genomic region was obtained from http://flybase.org/. All further characterizations were performed using the GENtle software.

#### qPCR

To test whether further Rh7 isoforms exist in *D. melanogaster*, qPCR analysis was performed. Total RNA was extracted from the retinas of 3–4 wildtype flies (strain *CantonS*) using the Quick-RNA™ MicroPrep Kit from Zymo Research and reversely transcribed using the Qiagen QuantiTect Reverse Transcription Kit. qPCRs were then carried out with the Bioline SensiFAST SYBR No-ROX Kit in combination with the Qiagen Rotor-Gene Q machine and 0.1 μM PCR primers. For each primer pair, three biological replicates were examined and for each replicate two qPCRs were run. The relative mRNA levels were calculated by using the ΔCT–equation and alpha-tubulin was used as the reference gene. The following genes and primers (sequences 5’–3’) were used:

Rh7 ex2-3: ACCACGACAAGCACGTGAATG; TGGCTCGAACTGTAGCCAAAAG Rh7 hyp(a): GGCTTATGAAGTTGCAAAAAGAATTC; TGGCTCGAACTGTAGCCAAAAG
Rh7 hyp(b): CAATACAATGTCAAATAGTTCTGAAACCA; TGGCTCGAACTGTAGCCAAAAG
Alpha-tub: TCTGCGATTCGATGGTGCCCTTAAC; GGATCGCACTTGACCATCTGGTTGGC

## Results

### Sequence homology

**Figure 2:**
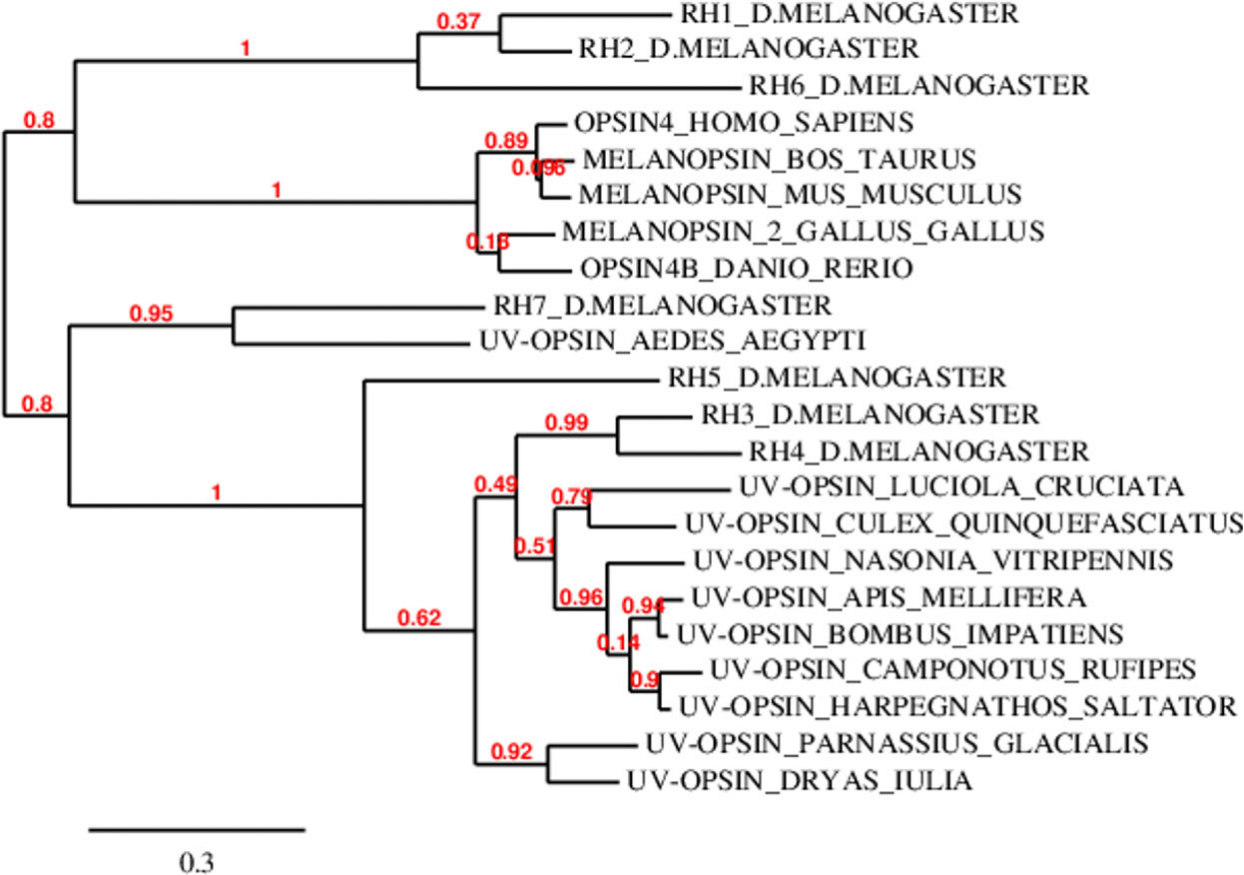
Phylogenic analysis of *Drosophila* Rhodopsins and other randomly chosen opsins from other animals. While Rh1, Rh2, and Rh6 form a cluster with vertebrate melanopsins, Rh3, Rh4, Rh5, and Rh7 form an insect specific cluster. The branch length is proportional to the number of substitutions per site. Branch support values are given as red numbers (with 1 as the highest value).

To inquire into the protein sequence conservation between Rh7 and all other Rhodopsins BLAST analyses with corresponding protein sequences were performed. This analysis revealed that the presumed Rh7 protein is closely related to other known *Drosophila* Rhodopsins. Expectation (e)-values are the most reliable and sensitive indicator of likely sequence homology. A similarity score of e < 0.01 or e < 0.001 simply says that this score should occur by chance once in 100 or once in 1,000 database searches, respectively (Pearson, 2016). Rh7 had the following e-values, when compared to the different Rhodospins: 1.7e^−46^ for Rh1, 7.2e^−49^ for Rh2, 4.9e^−58^ for Rh3, 2.9e^−55^ for Rh4, 7.8e^−52^ for Rh5, and 1.6e^−41^ for Rh6 were obtained and revealed that Rh3, Rh4, and Rh5 are the closest relatives of Rh7 (Table 1; Fig. 2). All three Rhodopsins displayed one common feature: they were all expressed in the inner receptor cells of the compound eye.

**Table 1:** Sequence identities between Rhodopsins in percentage. Pairwise comparison of amino acid sequences of Rhodopsins (http://imed.med.ucm.es/Tools/sias.html). Rhodopsins from the same group exhibit very strong identities about 70% and Rhodopsins from different groups exhibit about 30% identities. All *Drosophila* Rhodopsins are related to vertebrate Melanopsins and have amino acid identities of about 30%. However, Rh1, Rh2, and Rh6 are the most similar Rhodopsins to Melanopsin. Rh7 is the farthermost, when compared with *Bos taurus* melanopsin.

Dmel Rh1	100%								
Dmel Rh2	69.16%	100%							
Dmel Rh3	35.38%	34.38%	100%						
Dmel Rh4	35.12%	35.71%	73.01%	100%					
Dmel Rh5	32.17%	32.28%	40.83%	44.44%	100%				
Dmel Rh6	51.76%	51.21%	31.16%	32.79%	30.35%	100%			
Dmel Rh7	29.75%	28.87%	30.28%	30.42%	30.36%	27.1%	100%		
Btau Opsin1	24.71%	24.42%	22.7%	23.56%	22.41%	25%	23.27%	100%	
Btau Melanopsin	31.09%	30.44%	28.45%	29.89%	27.22%	31.7%	24.47%	25.57%	100%
	Dmel Rh1	Dmel Rh2	Dmel Rh3	Dmel Rh4	Dmel Rh5	Dmel Rh6	Dmel Rh7	Btau Opsin1	Btau Melanopsin

In the next step, the amino acid sequence of Rh7 was compared with all other *Drosophila* Rhodopsins as well as with the vertebrate Rhodopsin and Melanopsin of *Bos Taurus* (Table 1). The protein sequence of Rh7 is very similar to that of other *Drosophila* Rhodopsins (Fig. S1; Table 1): about 30% of Rh7 the amino acids were identical to amino acids of other *Drosophila* Rhodopsins (Rh1: 29.75%, Rh2: 28.87%, Rh3: 30.28%, Rh4: 30.42%, Rh5: 30.36%, and Rh6: 26.51%) and about 24% to vertebrate opsins (Rhodopsin: 23.27%, Melanopsin: 24.47%) (Table 1). Sequence identity between the other *Drosophila* Rhodopsins was variable and ranged from ∼30 to 69% (Table 1). Similarly to Rh7, the sequence identity between the other *Drosophila* Rhodopsins and vertebrate opsins was lower (between 22.4 and 31.1%) than within the *Drosophila* Rhodopsins.

### Phylogenetic analyses

Phylogenetic analysis of protein sequences of all *Drosophila* Rhodopsin yielded a tree with two large clades with Rh1, Rh2, and Rh6 forming the first cluster, and Rh3, Rh4, Rh5, and Rh7 forming the second cluster. Although all *Drosophila* Rhodopsins are related to vertebrate melanopsins, Rh1, Rh2, and Rh6 are the closest relatives, while Rh3, Rh4, and Rh5 are closer related to other insect-type opsins. Rh7, although in the same cluster with Rh3, Rh4, and Rh5, is outlying when compared with the other three Rhodopsins (Fig. 2).

**Figure 3:**
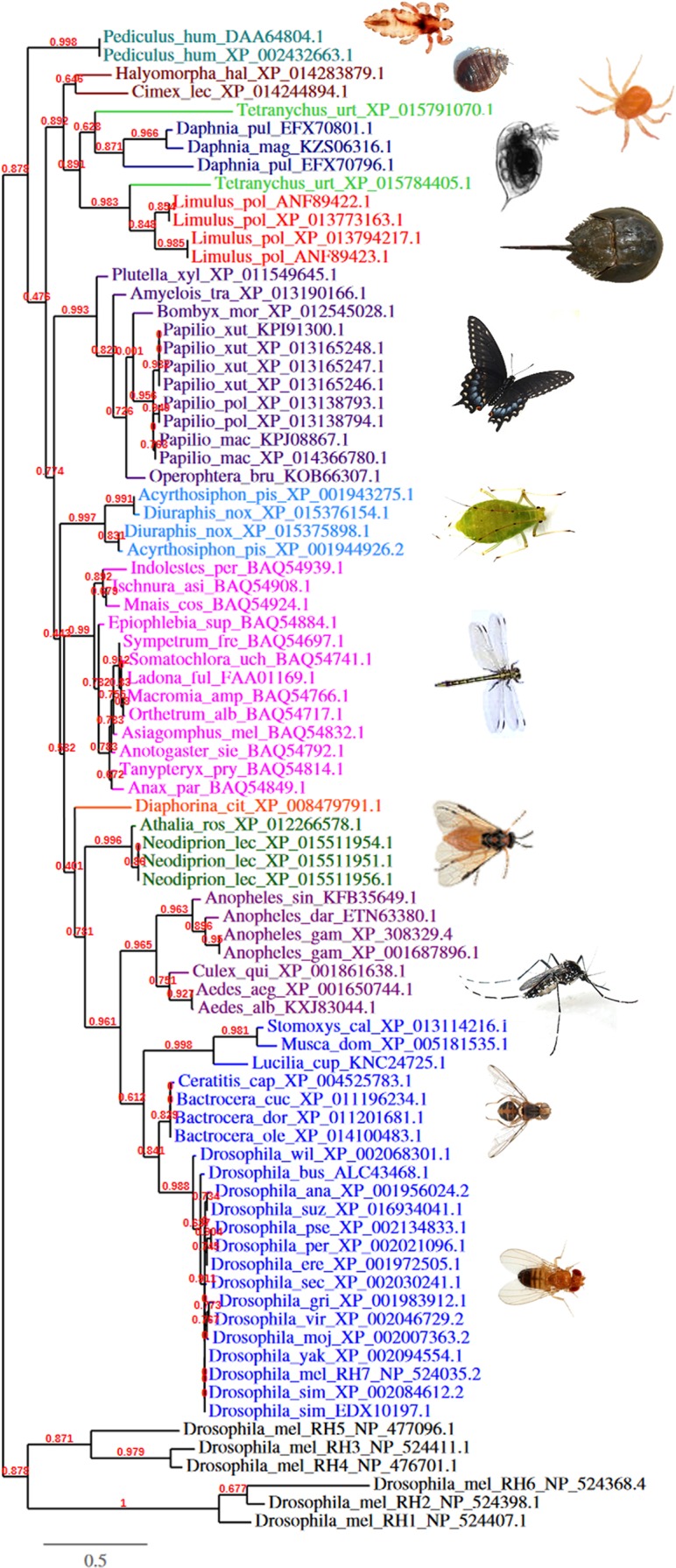
Phylogenetic analysis of all probable Rh7 homologues. Probable Rh7 homologues were obtained by BLAST analysis with http://blast.ncbi.nlm.nih.gov/Blast.cgi (Table S1). The branch length is proportional to the number of substitutions per site. Branch support values are given as red numbers. Only proteins that cluster indeed with Rh7 and not with other Rhodopsins are shown. The phylogenetic tree reveal that Rh7 and its homologues form a third Rhodopsin cluster, when compared with other *Drosophila* Rhodopsins.

To investigate the evolutionary role of Rh7, we searched for putative Rh7 homologues in all so far sequenced organisms. Putative Rh7 homologues were found in the phylum of arthropods. A second phylogenetic analysis with all presumptive Rh7 homologues (see Table S1) revealed that they are in the same lineage with Rh7. This suggests the presence of a third Rhodopsin cluster: the Rh7-group (Fig. 3). Rh7 homologues are namely present in water fleas (*Daphnia*), spider mites (*Tetranynchus*), bugs (*Cimex* and *Halyomorpha*), butterflies (*Papilio*), aphids (*Diuraphis* and *Acyrthosiphon*), dragonflies (*Anax*, *Orthertrum*, etc.), sawflies (*Athalia*, *Neodiprion*), mosquitos (*Anopheles*, *Aedes*, etc.), and ‘real’ fruit flies (*Ceratitis*, *Bactrocera*). Additionally, ancient animals like the atlantic horseshoe crab *Limulus polyphemus* own Rh7 homologues. Some animals (e.g. aphids and water fleas) even possess more than one Rh7 relative in their genome. Other insects, such as bees and ants belonging to the derived apocrita group of hymenoptereans may have lost Rh7 during evolution, since the more basic hymenopterean sawflies (symphyta) possess Rh7. However, none of the animals living in a pelagic, aphotic environment were found to possess Rh7.

### Structure of Rhodopsin 7

**Figure 4:**
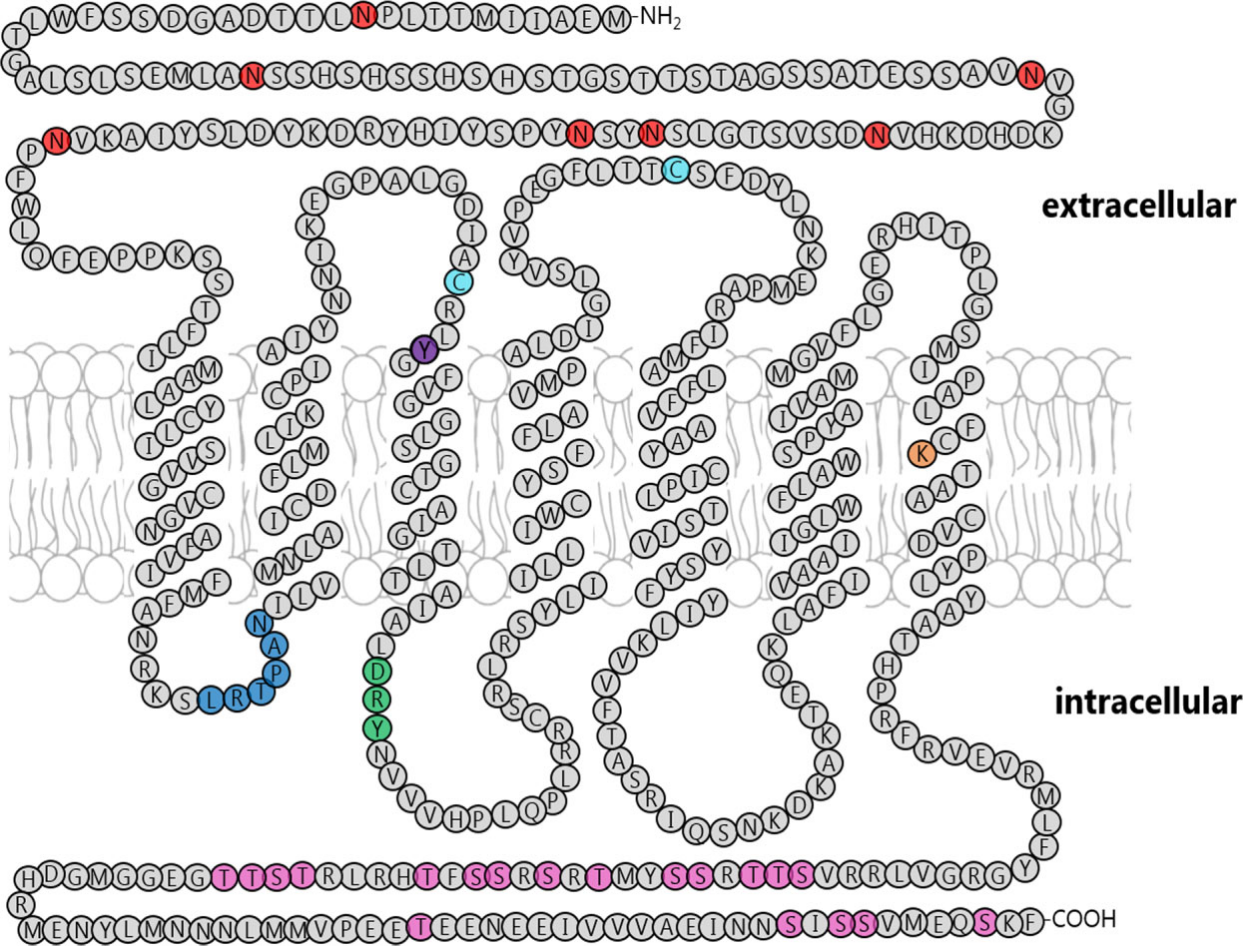
Tertiary structure of the presumptive Rh7 protein. The assumed Rh7 protein share several highly conserved features with other Rhodopsins. This includes: seven transmembrane domains, an extracellular N-Terminus, an intracellular C-Terminus, the chromophore binding lysine in the seventh transmembrane domain, the LRTPXN motif in the first cytoplasmic loop, the DRY motif in the second cytoplasmic loop, two cysteines (C) in the second and third extracellular loops, and a conserved tyrosine residue in the third transmembrane domain. However, Rh7 differs from other Rhodopsins with its extended N- and C-Termini and by the lack of the QAKK motif in the third cytoplasmic loop.

The tertiary structure of the assumed Rh7 protein is very similar to that of other Rhodopsins and contains several highly conserved features that are important for a functional protein (Figs. 4 and S1). Hydrophobicity analysis by PolyPhobius revealed that Rh7 possess seven transmembrane domains like all other Rhodopsins. Rhodopsins are characterized by an extracellular N-Terminus with many asparagines (N) that are glycosylated and an intracellular C-Terminus with several serines (S) and threonines (T) that serve as possible light-dependent phosphorylation sites for the Rhodopsin kinase (Ohguro et al., 1996; Papatsenko, Sheng & Desplan, 1997). Both tails are important for signal-transduction whereby the N-terminus tails of the different Rhodopsins differ in general very much (Fig. S3). Only the N-termini of Rh1 and Rh2, as well as the N-termini of Rh3 and Rh4 share about 50% similar amino acids. The lowest sequence similarity of 8% is found between the N-termini of Rh1 and Rh5. Rh7 has a sequence similarity of its N-terminus with the N-termini of the other Rhodopsins ranging between 12 and 22% (Fig. S3). Compared to other Rhodopsins, the N-terminus of Rh7 is very long: Rh1 has an N-terminus tail of about 50 amino acids. In Rh2 the tail contains 57 amino acids, in Rh3 58 amino acids, in Rh4 54 amino acids, in Rh5 50 amino acids, and in Rh6 45 amino acids. The N-terminus of Rh7 contains instead 115 amino acids and is therefore twice as long as the others. This is also true for the C-terminus: In Rh1 the C-terminus contains 42 amino acids, while in Rh2 it contains 43 amino acids, in Rh3 42 amino acids, in Rh4 41 amino acids, in Rh5 48 amino acids, and in Rh6 41 amino acids. In Rh7, the C-terminus is 96 amino acids long hence again twice as long as the others. The prolonged N- and C-termini lead Rh7 to possessing many more asparagines (N) in the N-terminus as well as serines (S) and threonines (T) in the C-terminus compared to other Rhodopsins.

The chromophore binding lysine (K)-residue is another important feature of all Rhodopsins. This highly conserved residue is bound via a Schiff’s base linkage by the retinal chromophore. Rh7 also possess this lysine, which is found at position 374 in the seventh transmembrane domain.

The LRTPXN motif in the first cytoplasmic loop, of which the leucine (L) and asparagine (N) are necessary for the formation of a functional Rhodopsin is also found in Rh7. Rh7 also owns the DRY motif in the second cytoplasmic loop. This motif is necessary for the interaction with the G-protein (Franke et al., 1992) as well as the two cysteines (C) in the second and third extracellular loop that form a disulfide bond (Maden, 1995). Rh1 possess a conserved tyrosine (Y) residue three amino acids apart from the first cysteine in the third transmembrane domain. This tyrosine can also be found in all other Rhodopsins that detect visible light i.e. Rh2, Rh5, and in Rh6, while this residue is changed by a phenylalanine (F) in UV-light sensitive Rh3 and Rh4 (Chou et al., 1996). In this position, Rh7 possesses a tyrosine like visible light detecting Rhodopsins, thus suggesting that Rh7 also respond to visible light.

**Figure 5:**
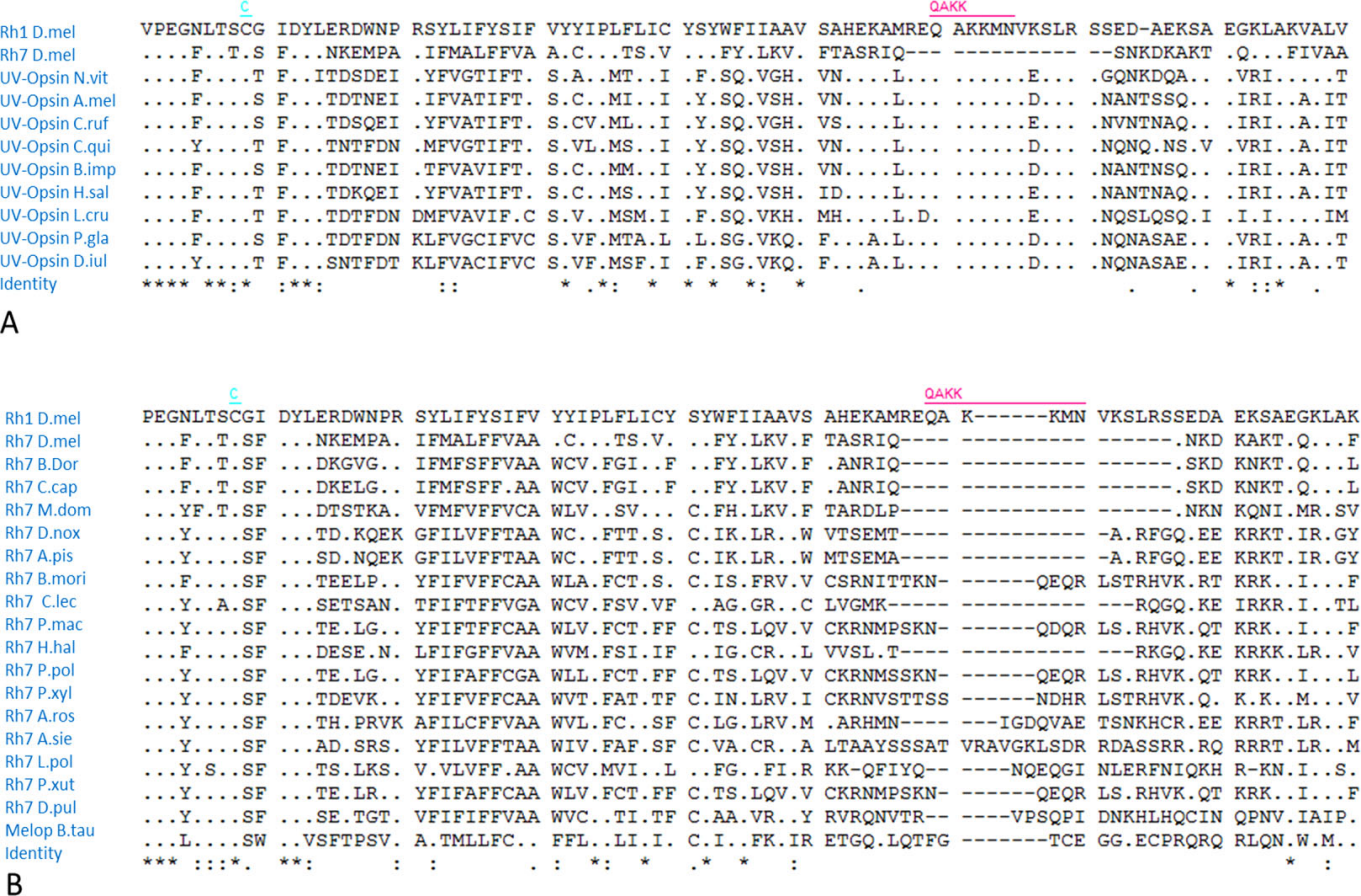
Multiple alignment of the amino acid sequence of diverse Opsin proteins. (A) The primary structure of known Opsins reveal that the QAKK domain is highly conserved among insect Opsins, but missing in *Drosophila* Rh7. (B) However all homologues of the *Drosophila* Rh7 as well as vertebrate melanopsins lack this motif.

The Rh7 sequence strongly differs from all other known insect rhodopsins in the third cytoplasmic loop. This part of the protein has the conserved QAKK motif, which mediates the interaction with the G-protein along with the DRY motif in the second cytoplasmic loop. Rh7 lacks the QAKK motif in its third cytoplasmic loop. Although the QAKK motif is highly conserved in many so far characterized insect opsins (Fig. 5A), it is absent in vertebrate opsins (e.g. the *Bos taurus* opsin), as well as in all insect opsins that cluster with *Drosophila* Rh7 (Fig. 5B).

### Rh7 locus

**Figure 6:**
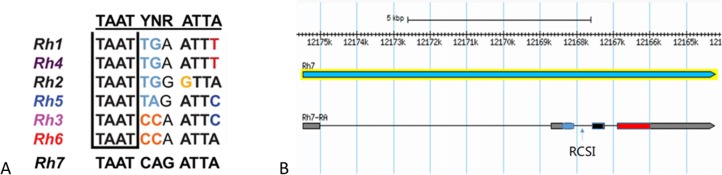
The cis-regulatory Rhodopsin Core Seqeunce I (RCSI) is highly conserved in the Rhodopsin gene locus. (A) Like all other *Drosophila* *Rhodopsin* genes, *Rh7* also possess the RCSI motif. (B) The RCSI motif of *Rh7* is located in the second intron of the *Rh7* gene and not 100 bps upstream from the transcription start site.

To search for further conserved Rhodopsin regions, we examined its genomic region. All *Drosophila* genes coding for proteins that are involved in the phototransduction cascade, such as Rhodopsins, G-proteins, arrestins, TRP and TRPL ion channels, scaffolding proteins, phospholipase C, etc. possess a conserved cis-regulatory element with the nucleotide sequence TAATYNRATTA, called RCSI, about 100 bps upstream of the transcription start site (Rister et al., 2015). While this sequence is palindromic in phototransduction genes that are broadly expressed in each photoreceptor cell, the last nucleotide differs in Rhodopsin genes, indicating their receptor cell-specific expression. An exception is *Rh6*, in which the ending is still “ATTA,” but the middle sequences differ from the broadly expressed phototransduction genes. *Rh7* also possesses a RCSI with the nucleotide sequence TAATCAGATTA (Fig. 6A) which is palindromic like that of broadly expressed phototransduction genes and thereby differs from other Rhodopsin genes. In addition, instead of being located 100 bps upstream of the transcription start site, the RCSI is found within the second intron of the *Rh7* gene (Fig. 6B).

To investigate whether *Rh7* may have lost its original function in the course of evolution and is no longer expressed in the photoreceptor cells of *Drosophila melanogaster*, which is suggested by the RCSI no longer appearing 100 bp before the transcription start site, we screened the genomic region of twelve other sequenced *Drosophila* species for the RCSI. Five out them (*D. melanogaster*, *D. simulans*, *D. sechellia*, *D. yakuba*, and *D. erecta*) possess the RCSI in the Rh7 gene locus. In *D. ananassae* the sequence seem to be bit modified and all other species (*D. pseudoobscura pseudoobscura*, *D. persimilis*, *D. willistoni*, *D. mojavensis*, *D. virilis*, and *D. grimshawi*) did not exhibit the RCSI. Thus, the expression pattern of *Rh7* might have indeed changed during evolution.

In all species that possess the RCSI, this motif is located in an intron of the *Rh7* gene and not upstream of the putative transcription start site. Most interestingly, the RCSI motif is always followed by the next exon starting with the coding sequence NYSNY and the Rh7 amino acid sequences of the different *Drosophila* species are highly conserved downstream of the NYSNY sequence, whereas they are highly variable upstream of it (Figs. S2 and S3). This suggests that the real transcription start site might not locate upstream but downstream the RCSI motif as in the other Rhodopsins. Indeed, there are two hypothetical translation start sites in the region downstream the RCSI. If translation starts at one of these sites, the N-terminus of Rh7 will be only ∼50 amino acids long and will show a higher homology to the N-termini of the other Rhodopsins. To test this possibility, we performed qPCR analysis on *D. melanogaster* retinas with primers in the region of the intron containing the RCSI motif. However, we obtained only PCR products for the previously described Rh7 isoform with the extended and non-conserved N-terminus and not for the hypothetical ones (Fig. S4). This does not only show that the shorter isoforms of Rh7 are not existent, but also that Rh7 with the long N-terminus tail is indeed expressed in the retina, suggesting that the RCSI is functional in *D. melanogaster*.

## Discussion

The six previously identified *Drosophila* Rhodopsins fall into two groups: the vertebrate-melanopsin-type-opsins, and the insect-type-opsins. Whereas Rh3, Rh4, and Rh5 are very similar to other insect-opsins (e.g. UV-opsin *Apis mellifera*), Rh1, Rh2, and Rh6 are more closely related to vertebrate-melanopsins. The sequence similarity between Rhodopsins belonging to the same group is very high. Rh1 shares 69.16% of its amino acids with Rh2, and 51.65% with Rh6, while Rh3 is 73.01% identical to Rh4 and 40.83% to Rh5.

However, Rh7 only shares about 30% of its amino acids with other *Drosophila* Rhodopsins. This seems to be a small overlap compared to other Rhodopsin identities from the same group. However, even members from the two known groups only share 30–35% of their amino acids (Rh1, Rh2, and Rh6 compared with Rh3, Rh4, and Rh5). This already indicates that Rh7 might belong to a third, so far uncharacterized, Rhodopsin group.

Phylogenic tree analyses support the idea that Rh7 might belong to a novel Rhodopsin group. As expected Rh1, Rh2, and Rh6 cluster together with vertebrate melanopsins, while Rh3, Rh4, and Rh5 cluster with other insect opsins. Rh7 though, despite related to the insect-type-opsin group, seems to form its own unique group: the “Rh7-like” group. The fact that Rh7 homologues are already present in ancient animals like *Limulus polyphemus* and *Daphnia pulex* indicates that the *Rh7* gene is indeed a conserved protein coding gene, otherwise random mutations would have appeared in the coding region of the gene. However, like all *Rhodopsin* genes, the *Rh7* gene also seems to be duplicated in some animals. Both aphids as well as *Daphnia* possess more than one *Rh7* relative in their genome, yet whether these multiple replicates are real protein coding genes or merely pseudogenes remains to be studied. Although many different arthropods possess Rh7 homologues, hymenopterans belonging to the group of apocrita for example don’t possess Rh7 homologues. Since *Athalia rosae* and *Neodiprion lecontei* having its place within the symphyta (sawflies), the more basic group of hymenoptera, still possess Rh7, it is likely that the apocrita may have lost Rh7 during evolution due to different habituation.

Although the protein structure of Rh7 is very similar to all other known Rhodopsins, it slightly differs from the other *Drosophila* Rhodopsins. With its prolonged N- and C-termini, Rh7 possesses many more asparagines (N), serines (S) and threonines (T) than other Rhodopsins do. All these amino acids are important for protein-protein-interaction, thus Rh7 may interact with more and other interaction partners as compared to the known *Drosophila* Rhodopsins. In addition, it is notable that the N-termini of all *Drosophila* Rhodopsins differ a lot, when compared to each other (Fig. S3). This suggests that the N-terminus of each Rhodopsin has unique properties. Rh7 has the most conspicuous N-terminus. It is not only long, but has also the highest potency to undergo interactions with other proteins, perhaps even with other Rhodopsins. G-protein-coupled receptors (GPCRs) including Rhodopsin-like GPCRs can form dimers (Gurevich & Gurevich, 2008a; Gurevich & Gurevich, 2008b; Hiller, Kühhorn & Gmeiner, 2013). This dimerization seems not necessary for G-protein binding and activation, but it seems to influence signaling in the phototransduction cascade (White et al., 2007).

The assumption that Rh7 may interact with another Rhodopsin to exert its function is supported by the fact that Rh7 and its homologues miss the highly conserved QAKK motif, which is, together with the DRY motif, important for G-protein binding and consequently for the activation of the G-protein-coupled cascade. Thus, Rh7 might signal via a second Rhodopsin and not by itself. The presence of more than one Rhodopsin in one receptor cell has been shown in vertebrates and invertebrates (Applebury et al., 2000; Hu et al., 2014; Mazzoni, Desplan & Çelik, 2004; Stavenga & Arikawa, 2008) and makes this possible. Nevertheless, it is also imaginable that the conserved DRY motif alone, that is present in Rh7, suffices for G-protein binding. The latter is suggested by a study in the mosquito *Aedes aegypti* (Hu et al., 2014) showing that the Rh7 homologue of the mosquito, Op10, is a functional Rhodopsin although it lacks the QAKK motif. Op10 can even activate the phototransduction cascade in R1–6 of *D. melanogaster*. This suggests that G-protein binding is still possible even in the absence of the QAKK motif, but it does not exclude the possibility that Rh7 dimerizes with a second Rhodopsin and by this way affects signaling.

The distinctiveness of Rh7 is also confirmed by its genomic structure: first it possesses a palindromic RCSI, which is atypical for Rhodopsins, and, second, it possesses the RCSI in the middle of the gene while all other Rhodopsin genes possess the RCSI about 100 bp upstream of the transcription starting site. The hypothesis that the crucial role of Rh7 may have been lost in *D. melanogaster* during evolution, but may still present in other *Drosophila* species living in different habitats, is unsupported by the fact that some of the tested *Drosophila* species don’t even own the RCSI in their genomic locus. These results might indicate that Rh7 is differently activated by other transcription factors than other Rhodopsin genes and that it could therefore be expressed in other tissues and developmental stages than other known Rhodopsins. Nevertheless, our qPCR results show that Rh7 with the long N-terminus tail is expressed in the retina of *D. melanogaster*.

In summary, we show here that Rh7 is a peculiar Rhodopsin with unusual properties. Its presence in most groups of arthropods, excluding those living in aphotic environments, suggests that it has a conserved function in light-signaling either as visual or non-visual Rhodopsin. Rh7 might play a role in basic insect circadian photoreception and thus play a similar role in insects as melanopsin plays in mammals. It will be most promising to unravel this putative function of Rh7 by using the arsenal of genetic techniques available in *D. melanogaster*. We are currently pursuing this endeavor.

## Conclusion

Rh7 seems to be an ancient type of Rhodopsin, which is conserved among different arthropods but lost in other. Rh7 possess nearly all important features for a functional visual protein, but it differs from other known Rhodopsins in its genomic characteristics, suggesting that Rh7 may have a different role than other known Rhodopsins. The palindromic RCSI in the first intron, which is even lost in some species suggests that *Rh7* is transcribed differently from other Rhodopsins. In addition, the structural properties of Rh7 suggest that it might undergo different pathways compared to all other known Rhodopsins.

## Supplemental Information

10.7717/peerj.2427/supp-1 Supplemental Information 1 Supplemental Information.

10.7717/peerj.2427/supp-2 Supplemental Information 2 Multiple alignments of all *Drosophila* Rhodopsins. The transmembrane domains are shown in grey, the LRTPXN motif is shown in blue, the disulfide bridge binding cysteines are shown in cyan, the visual light sensitive tyrosine is shown purple, the DRY motif is shown in green, the chromophore binding lysine is shown in orange, and the proper location of the QAKK motif is shown magenta.

10.7717/peerj.2427/supp-3 Supplemental Information 3 Multiple alignments of Rh7 proteins from different *Drosophila* species.

10.7717/peerj.2427/supp-4 Supplemental Information 4 Multiple alignments of N-termini of all Drosophila Rhodopsins. The N-termini of all *Drosophila* Rhodopsins were aligned. The amino acid sequences before the first transmembrane domain starts were extracted for the alignment. The N-termini of each Rhodopsin seem to be kept very variable.

10.7717/peerj.2427/supp-5 Supplemental Information 5 qPCR analysis for hypothetically possible isoforms using WT CantonS retinas.

10.7717/peerj.2427/supp-6 Supplemental Information 6 List of all presumptive Rh7 homologues. BLAST analysis was performed in http://blast.ncbi.nlm.nih.gov/Blast.cgi?PAGE=Proteins using the Rh7 protein sequence.

10.7717/peerj.2427/supp-7 Supplemental Information 7 Sequence identities between N-termini of all *Drosophila* Rhodopsins in percentage. Pairwise comparison of amino acid sequences of Rhodopsins (http://imed.med.ucm.es/Tools/sias.html). The N-terminus of all Rhodopsins, with the exception of Rh1/Rh2 and Rh3/Rh4, differ a lot, while the whole protein identities (Table 1) are at least about 30% similar.

## Additional Information and Declarations

### Data Deposition

The following information was supplied regarding data availability: All *Drosophila* Rhodopsin sequences were obtained from Flybase (www.flybase.org; FB2016_02, released March 30, 2016).
