# Supplementary material for: Rhodopsin 7–The unusual Rhodopsin in Drosophila
Source: PeerJ. 2016 Sep 6;4:e2427. doi: 10.7717/peerj.2427 (PMC5018682; doi:10.7717/peerj.2427)
Supplement: Supplemental Information 1 [file peerj-04-2427-s001.docx]

## Supplemental Information


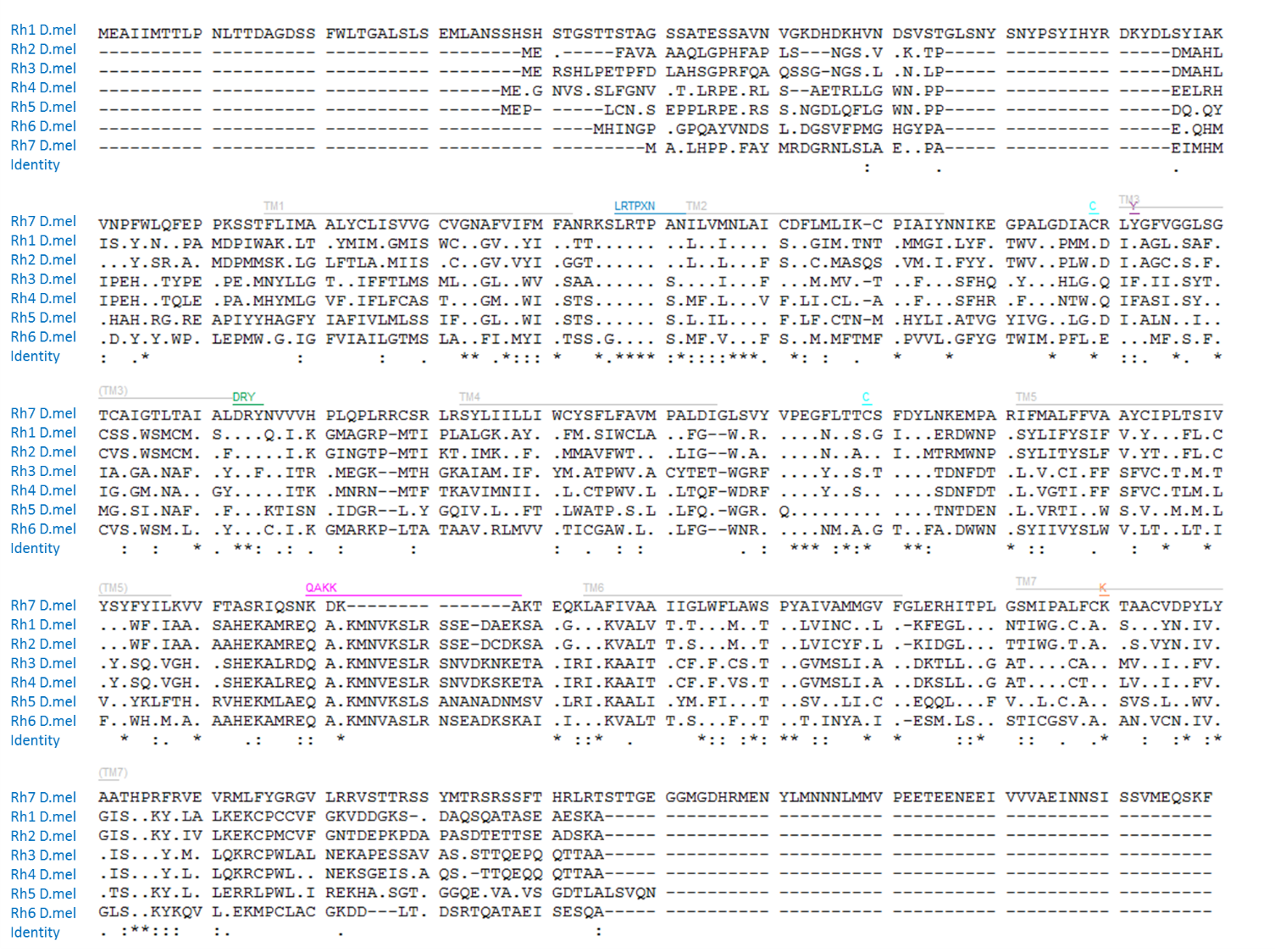


Figure S 1

**Multiple alignments of all *Drosophila* Rhodopsins.** The transmembrane domains are shown in grey, the LRTPXN motif is shown in blue, the disulfide bridge binding cysteines are shown in cyan, the visual light sensitive tyrosine is shown purple, the DRY motif is shown in green, the chromophore binding lysine is shown in orange, and the proper location of the QAKK motif is shown magenta.


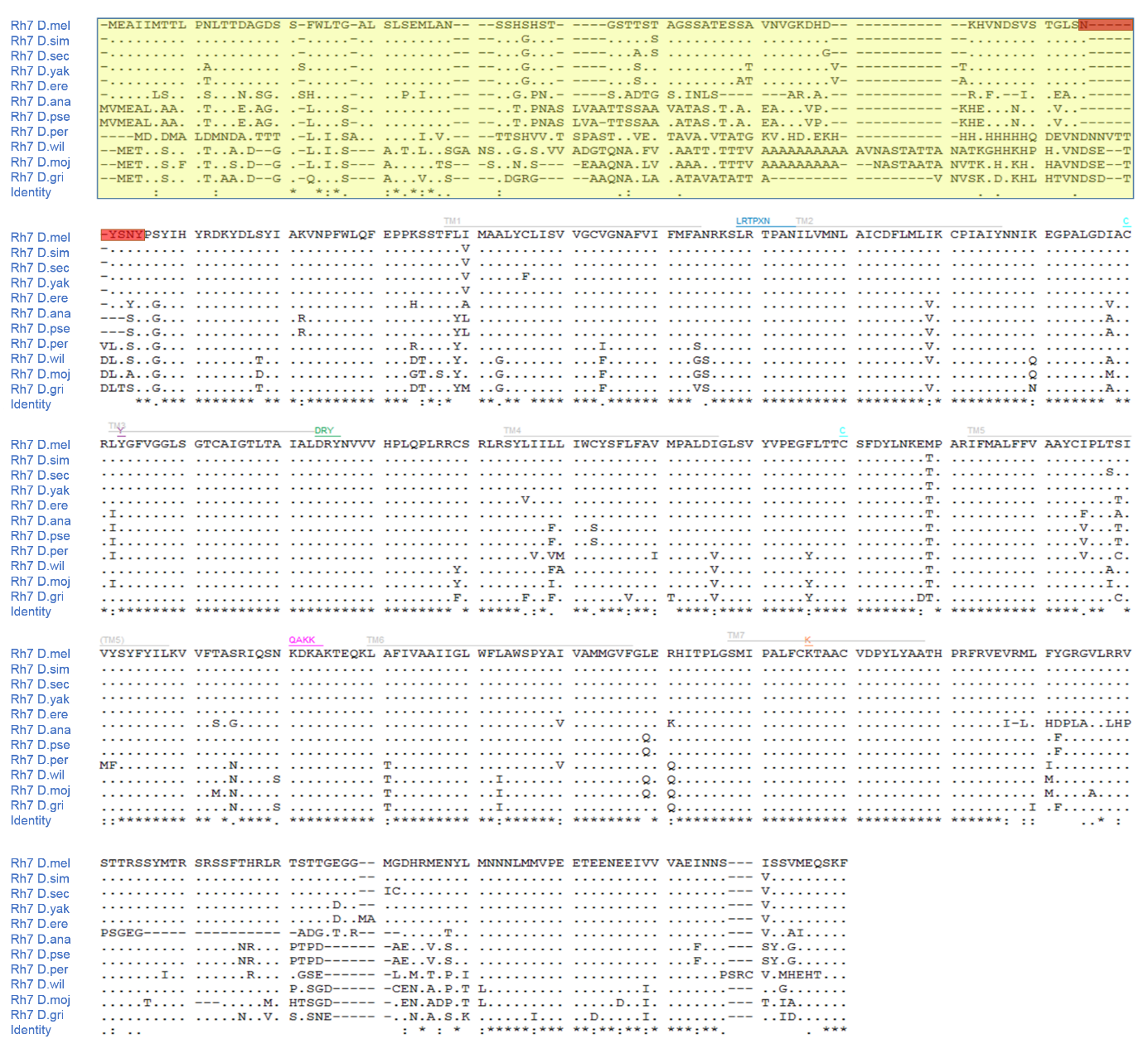


Figure S 2

**Multiple alignments of Rh7 proteins from different *Drosophila* species.**

Rh7 amino acid sequences from different *Drosophila* species vary a lot in front of (yellow) and are highly conserved after the NYSNY sequence (red bar). The Rh7 sequence of *Drosophila simulans* was modified, since it seemed to have a prediction error. The prior predicted protein sequence would have a 70 amino acids long overhang, which is not found in any other *Drosophila* species.


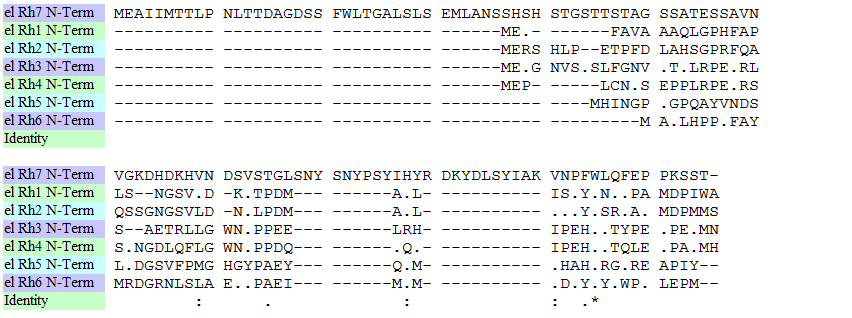


Rh7 N-Term

Rh1 N-Term

Rh2 N-Term

Rh3 N-Term

Rh4 N-Term

Rh5 N-Term

Rh6 N-Term

Identity

Rh7 N-Term

Rh1 N-Term

Rh2 N-Term

Rh3 N-Term

Rh4 N-Term

Rh5 N-Term

Rh6 N-Term

Identity

Figure S 3

**Multiple alignments of N-termini of all Drosophila Rhodopsins.**

The N-termini of all *Drosophila* Rhodopsins were aligned. The amino acid sequences before the first transmembrane domain starts were extracted for the alignment. The N-termini of each Rhodopsin seem to be kept very variable.


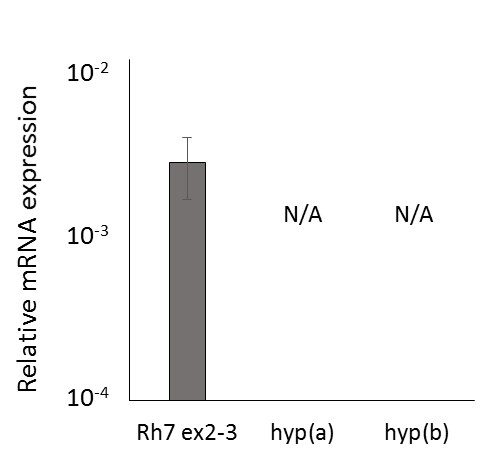


Figure S 4

**qPCR analysis for hypothetically possible isoforms using WT CantonS retinas**. In all other Rhodopsins, the RCSI is located upstream the coding region and transcription starts approximately 100bp after the RCSI. Only in *Rh7*, the RCSI is located in the intron between exon 2 and 3 and transcription seems to start 7.5 kb upstream of the RCSI giving rise to a very long N-Terminus of the Rh7 protein. Since there are two putative additional translation start sites downstream of the RCSI of Rh7, two additional isoforms of Rh7 may exist. To check whether these *Rh7* isoforms are present, we used primer sequences in the intron between exon2 and exon3 and as controls primers spanning exon 2 and 3. PCR only revealed PCR products for the previously described *Rh7* isoform coding for protein with the extended and non-conserved N-Terminus and not for the hypothetical ones (hyp(a) and hyp(b), Figure S 3).

| Accession | Organism | Query cover | E value | Ident |
| --- | --- | --- | --- | --- |
| [NP_524035.2](http://www.ncbi.nlm.nih.gov/protein/24663181?report=genbank&log$=prottop&blast_rank=1&RID=P7CNM1K0015) | *Drosophila melanogaster* | 100% | 0% | 100% |
| [XP_002084612.2](http://www.ncbi.nlm.nih.gov/protein/1013917308?report=genbank&log$=prottop&blast_rank=2&RID=P7CNM1K0015) | *Drosophila simulans* | 100% | 0% | 99% |
| [EDX10197.1](http://www.ncbi.nlm.nih.gov/protein/194196621?report=genbank&log$=prottop&blast_rank=3&RID=P7CNM1K0015) | *Drosophila simulans* | 100% | 0% | 99% |
| [XP_002030241.1](http://www.ncbi.nlm.nih.gov/protein/195327063?report=genbank&log$=prottop&blast_rank=4&RID=P7CNM1K0015) | *Drosophila sechellia* | 100% | 0% | 98% |
| [XP_001972505.1](http://www.ncbi.nlm.nih.gov/protein/194869710?report=genbank&log$=prottop&blast_rank=5&RID=P7CNM1K0015) | *Drosophila erecta* | 100% | 0% | 97% |
| [XP_002094554.1](http://www.ncbi.nlm.nih.gov/protein/195493766?report=genbank&log$=prottop&blast_rank=6&RID=P7CNM1K0015) | *Drosophila yakuba* | 100% | 0% | 98% |
| [XP_016934041.1](http://www.ncbi.nlm.nih.gov/protein/1036061592?report=genbank&log$=prottop&blast_rank=7&RID=P7CNM1K0015) | *Drosophila suzukii* | 100% | 0% | 95% |
| [XP_001956024.2](http://www.ncbi.nlm.nih.gov/protein/964135588?report=genbank&log$=prottop&blast_rank=8&RID=P7CNM1K0015) | *Drosophila ananassae* | 100% | 0% | 86% |
| [XP_002134833.1](http://www.ncbi.nlm.nih.gov/protein/198464743?report=genbank&log$=prottop&blast_rank=9&RID=P7CNM1K0015) | *Drosophila pseudoobscura pseudoobscura* | 100% | 0% | 85% |
| [XP_002021096.1](http://www.ncbi.nlm.nih.gov/protein/195160465?report=genbank&log$=prottop&blast_rank=10&RID=P7CNM1K0015) | *Drosophila persimilis* | 100% | 0% | 85% |
| [XP_001983912.1](http://www.ncbi.nlm.nih.gov/protein/195013820?report=genbank&log$=prottop&blast_rank=11&RID=P7CNM1K0015) | *Drosophila grimshawi* | 99% | 0% | 80% |
| [XP_002046729.2](http://www.ncbi.nlm.nih.gov/protein/968115898?report=genbank&log$=prottop&blast_rank=12&RID=P7CNM1K0015) | *Drosophila virilis* | 99% | 0% | 78% |
| [XP_002007363.2](http://www.ncbi.nlm.nih.gov/protein/968041259?report=genbank&log$=prottop&blast_rank=13&RID=P7CNM1K0015) | *Drosophila mojavensis* | 99% | 0% | 81% |
| [XP_002068301.1](http://www.ncbi.nlm.nih.gov/protein/195440958?report=genbank&log$=prottop&blast_rank=14&RID=P7CNM1K0015) | *Drosophila willistoni* | 95% | 0% | 79% |
| [XP_011196234.1](http://www.ncbi.nlm.nih.gov/protein/751443117?report=genbank&log$=prottop&blast_rank=15&RID=P7CNM1K0015) | *Bactrocera cucurbitae* | 83% | 0% | 66% |
| [XP_011201681.1](http://www.ncbi.nlm.nih.gov/protein/751784747?report=genbank&log$=prottop&blast_rank=16&RID=P7CNM1K0015) | *Bactrocera dorsalis* | 83% | 0% | 66% |
| [ALC43468.1](http://www.ncbi.nlm.nih.gov/protein/924558456?report=genbank&log$=prottop&blast_rank=17&RID=P7CNM1K0015) | *Drosophila busckii* | 57% | 2,00E-169 | 88% |
| [XP_014100483.1](http://www.ncbi.nlm.nih.gov/protein/929380214?report=genbank&log$=prottop&blast_rank=18&RID=P7CNM1K0015) | *Bactrocera oleae* | 70% | 4,00E-161 | 68% |
| [KNC24725.1](http://www.ncbi.nlm.nih.gov/protein/906462480?report=genbank&log$=prottop&blast_rank=19&RID=P7CNM1K0015) | *Lucilia cuprina* | 79% | 1,00E-137 | 54% |
| [XP_004525783.1](http://www.ncbi.nlm.nih.gov/protein/498966319?report=genbank&log$=prottop&blast_rank=20&RID=P7CNM1K0015) | *Ceratitis capitata* | 61% | 2,00E-135 | 67% |
| [XP_013114216.1](http://www.ncbi.nlm.nih.gov/protein/907728888?report=genbank&log$=prottop&blast_rank=21&RID=P7CNM1K0015) | *Stomoxys calcitrans* | 73% | 6,00E-126 | 55% |
| [XP_005181535.1](http://www.ncbi.nlm.nih.gov/protein/557763648?report=genbank&log$=prottop&blast_rank=22&RID=P7CNM1K0015) | *Musca domestica* | 84% | 2,00E-125 | 48% |
| [KFB35649.1](http://www.ncbi.nlm.nih.gov/protein/668445239?report=genbank&log$=prottop&blast_rank=23&RID=P7CNM1K0015) | *Anopheles sinensis* | 72% | 2,00E-119 | 51% |
| [XP_308329.4](http://www.ncbi.nlm.nih.gov/protein/158285471?report=genbank&log$=prottop&blast_rank=24&RID=P7CNM1K0015) | *Anopheles gambiae str. PEST* | 67% | 2,00E-118 | 52% |
| [XP_015375898.1](http://www.ncbi.nlm.nih.gov/protein/985387628?report=genbank&log$=prottop&blast_rank=25&RID=P7CNM1K0015) | *Diuraphis noxia* | 69% | 2,00E-113 | 47% |
| [XP_001944926.2](http://www.ncbi.nlm.nih.gov/protein/328719619?report=genbank&log$=prottop&blast_rank=26&RID=P7CNM1K0015) | *Acyrthosiphon pisum* | 68% | 1,00E-109 | 46% |
| [XP_001650744.1](http://www.ncbi.nlm.nih.gov/protein/157109598?report=genbank&log$=prottop&blast_rank=27&RID=P7CNM1K0015) | *Aedes aegypti* | 66% | 5,00E-107 | 49% |
| [XP_001943275.1](http://www.ncbi.nlm.nih.gov/protein/193615573?report=genbank&log$=prottop&blast_rank=28&RID=P7CNM1K0015) | *Acyrthosiphon pisum* | 79% | 6,00E-106 | 42% |
| [XP_012545028.1](http://www.ncbi.nlm.nih.gov/protein/827544844?report=genbank&log$=prottop&blast_rank=29&RID=P7CNM1K0015) | *Bombyx mori* | 65% | 2,00E-105 | 50% |
| [XP_015376154.1](http://www.ncbi.nlm.nih.gov/protein/985421486?report=genbank&log$=prottop&blast_rank=30&RID=P7CNM1K0015) | *Diuraphis noxia* | 71% | 3,00E-105 | 45% |
| [XP_014244894.1](http://www.ncbi.nlm.nih.gov/protein/939250651?report=genbank&log$=prottop&blast_rank=31&RID=P7CNM1K0015) | *Cimex lectularius* | 73% | 6,00E-105 | 46% |
| [BAQ54908.1](http://www.ncbi.nlm.nih.gov/protein/761599219?report=genbank&log$=prottop&blast_rank=32&RID=P7CNM1K0015) | *Ischnura asiatica* | 73% | 8,00E-103 | 47% |
| [XP_013190166.1](http://www.ncbi.nlm.nih.gov/protein/913318290?report=genbank&log$=prottop&blast_rank=33&RID=P7CNM1K0015) | *Amyelois transitella* | 69% | 2,00E-102 | 46% |
| [XP_014366780.1](http://www.ncbi.nlm.nih.gov/protein/943970148?report=genbank&log$=prottop&blast_rank=34&RID=P7CNM1K0015) | *Papilio machaon* | 68% | 4,00E-102 | 47% |
| [XP_001861638.1](http://www.ncbi.nlm.nih.gov/protein/170051159?report=genbank&log$=prottop&blast_rank=35&RID=P7CNM1K0015) | *Culex quinquefasciatus* | 57% | 3,00E-101 | 55% |
| [DAA64804.1](http://www.ncbi.nlm.nih.gov/protein/856116597?report=genbank&log$=prottop&blast_rank=36&RID=P7CNM1K0015) | *Pediculus humanus corporis* | 71% | 7,00E-101 | 44% |
| [XP_014283879.1](http://www.ncbi.nlm.nih.gov/protein/939675733?report=genbank&log$=prottop&blast_rank=37&RID=P7CNM1K0015) | *Halyomorpha halys* | 68% | 9,00E-101 | 47% |
| [KXJ83044.1](http://www.ncbi.nlm.nih.gov/protein/1000213647?report=genbank&log$=prottop&blast_rank=38&RID=P7CNM1K0015) | *Aedes albopictus* | 59% | 6,00E-100 | 51% |
| [XP_013165246.1](http://www.ncbi.nlm.nih.gov/protein/910324350?report=genbank&log$=prottop&blast_rank=39&RID=P7CNM1K0015) | *Papilio xuthus* | 68% | 1,00E-99 | 46% |
| [XP_015511954.1](http://www.ncbi.nlm.nih.gov/protein/998503670?report=genbank&log$=prottop&blast_rank=40&RID=P7CNM1K0015) | *Neodiprion lecontei* | 64% | 2,00E-99 | 49% |
| [XP_015511956.1](http://www.ncbi.nlm.nih.gov/protein/998503672?report=genbank&log$=prottop&blast_rank=41&RID=P7CNM1K0015) | *Neodiprion lecontei* | 64% | 3,00E-99 | 49% |
| [ETN63380.1](http://www.ncbi.nlm.nih.gov/protein/568254415?report=genbank&log$=prottop&blast_rank=42&RID=P7CNM1K0015) | *Anopheles darlingi* | 62% | 3,00E-99 | 51% |
| [XP_015511951.1](http://www.ncbi.nlm.nih.gov/protein/998503664?report=genbank&log$=prottop&blast_rank=43&RID=P7CNM1K0015) | *Neodiprion lecontei* | 61% | 3,00E-99 | 51% |
| [FAA01169.1](http://www.ncbi.nlm.nih.gov/protein/761600095?report=genbank&log$=prottop&blast_rank=44&RID=P7CNM1K0015) | *Ladona fulva* | 71% | 1,00E-98 | 45% |
| [XP_013138793.1](http://www.ncbi.nlm.nih.gov/protein/909566808?report=genbank&log$=prottop&blast_rank=45&RID=P7CNM1K0015) | *Papilio polytes* | 68% | 1,00E-98 | 46% |
| [BAQ54717.1](http://www.ncbi.nlm.nih.gov/protein/761598532?report=genbank&log$=prottop&blast_rank=46&RID=P7CNM1K0015) | *Orthetrum albistylum* | 63% | 2,00E-98 | 48% |
| [BAQ54849.1](http://www.ncbi.nlm.nih.gov/protein/761598865?report=genbank&log$=prottop&blast_rank=47&RID=P7CNM1K0015) | *Anax parthenope* | 86% | 5,00E-98 | 39% |
| [BAQ54884.1](http://www.ncbi.nlm.nih.gov/protein/761599033?report=genbank&log$=prottop&blast_rank=48&RID=P7CNM1K0015) | *Epiophlebia superstes* | 63% | 8,00E-98 | 48% |
| [XP_011549645.1](http://www.ncbi.nlm.nih.gov/protein/768417961?report=genbank&log$=prottop&blast_rank=49&RID=P7CNM1K0015) | *Plutella xylostella* | 74% | 8,00E-98 | 43% |
| [BAQ54939.1](http://www.ncbi.nlm.nih.gov/protein/761599391?report=genbank&log$=prottop&blast_rank=50&RID=P7CNM1K0015) | *Indolestes peregrinus* | 79% | 2,00E-97 | 42% |
| [XP_012266578.1](http://www.ncbi.nlm.nih.gov/protein/817087588?report=genbank&log$=prottop&blast_rank=51&RID=P7CNM1K0015) | *Athalia rosae* | 62% | 3,00E-97 | 50% |
| [BAQ54814.1](http://www.ncbi.nlm.nih.gov/protein/761598795?report=genbank&log$=prottop&blast_rank=52&RID=P7CNM1K0015) | *Tanypteryx pryeri* | 80% | 1,00E-96 | 42% |
| [BAQ54741.1](http://www.ncbi.nlm.nih.gov/protein/761598652?report=genbank&log$=prottop&blast_rank=53&RID=P7CNM1K0015) | *Somatochlora uchidai* | 65% | 8,00E-96 | 48% |
| [BAQ54697.1](http://www.ncbi.nlm.nih.gov/protein/761598310?report=genbank&log$=prottop&blast_rank=54&RID=P7CNM1K0015) | *Sympetrum frequens* | 76% | 1,00E-95 | 43% |
| [BAQ54792.1](http://www.ncbi.nlm.nih.gov/protein/761598753?report=genbank&log$=prottop&blast_rank=55&RID=P7CNM1K0015) | *Anotogaster sieboldii* | 63% | 2,00E-95 | 48% |
| [BAQ54766.1](http://www.ncbi.nlm.nih.gov/protein/761598701?report=genbank&log$=prottop&blast_rank=56&RID=P7CNM1K0015) | *Macromia amphigena* | 65% | 9,00E-94 | 47% |
| [XP_013165248.1](http://www.ncbi.nlm.nih.gov/protein/910324354?report=genbank&log$=prottop&blast_rank=57&RID=P7CNM1K0015) | *Papilio xuthus* | 63% | 2,00E-92 | 47% |
| [BAQ54832.1](http://www.ncbi.nlm.nih.gov/protein/761598831?report=genbank&log$=prottop&blast_rank=58&RID=P7CNM1K0015) | *Asiagomphus melaenops* | 61% | 4,00E-92 | 47% |
| [XP_013794217.1](http://www.ncbi.nlm.nih.gov/protein/926606621?report=genbank&log$=prottop&blast_rank=59&RID=P7CNM1K0015) | *Limulus polyphemus* | 69% | 2,00E-91 | 42% |
| [ANF89423.1](http://www.ncbi.nlm.nih.gov/protein/1031182501?report=genbank&log$=prottop&blast_rank=60&RID=P7CNM1K0015) | *Limulus polyphemus* | 69% | 3,00E-91 | 42% |
| [XP_002432663.1](http://www.ncbi.nlm.nih.gov/protein/242024495?report=genbank&log$=prottop&blast_rank=61&RID=P7CNM1K0015) | *Pediculus humanus corporis* | 63% | 4,00E-91 | 45% |
| [XP_001687896.1](http://www.ncbi.nlm.nih.gov/protein/158285473?report=genbank&log$=prottop&blast_rank=62&RID=P7CNM1K0015) | *Anopheles gambiae str. PEST* | 52% | 3,00E-89 | 53% |
| [ANF89422.1](http://www.ncbi.nlm.nih.gov/protein/1031182499?report=genbank&log$=prottop&blast_rank=63&RID=P7CNM1K0015) | *Limulus polyphemus* | 72% | 1,00E-88 | 41% |
| [XP_013773163.1](http://www.ncbi.nlm.nih.gov/protein/926616116?report=genbank&log$=prottop&blast_rank=64&RID=P7CNM1K0015) | *Limulus polyphemus* | 68% | 2,00E-88 | 42% |
| [KPI91300.1](http://www.ncbi.nlm.nih.gov/protein/930648952?report=genbank&log$=prottop&blast_rank=65&RID=P7CNM1K0015) | *Papilio xuthus* | 59% | 5,00E-88 | 47% |
| [KOB66307.1](http://www.ncbi.nlm.nih.gov/protein/914557755?report=genbank&log$=prottop&blast_rank=66&RID=P7CNM1K0015) | *Operophtera brumata* | 69% | 9,00E-87 | 41% |
| [XP_013165247.1](http://www.ncbi.nlm.nih.gov/protein/910324352?report=genbank&log$=prottop&blast_rank=67&RID=P7CNM1K0015) | *Papilio xuthus* | 58% | 9,00E-87 | 48% |
| [XP_013138794.1](http://www.ncbi.nlm.nih.gov/protein/909566810?report=genbank&log$=prottop&blast_rank=68&RID=P7CNM1K0015) | *Papilio polytes* | 59% | 3,00E-86 | 47% |
| [XP_015791070.1](http://www.ncbi.nlm.nih.gov/protein/1005966130?report=genbank&log$=prottop&blast_rank=69&RID=P7CNM1K0015) | *Tetranychus urticae* | 62% | 1,00E-85 | 45% |
| [XP_015784405.1](http://www.ncbi.nlm.nih.gov/protein/1005952087?report=genbank&log$=prottop&blast_rank=70&RID=P7CNM1K0015) | *Tetranychus urticae* | 65% | 5,00E-83 | 41% |
| [EFX70801.1](http://www.ncbi.nlm.nih.gov/protein/321459751?report=genbank&log$=prottop&blast_rank=71&RID=P7CNM1K0015) | *Daphnia pulex* | 64% | 6,00E-81 | 42% |
| [EFX70796.1](http://www.ncbi.nlm.nih.gov/protein/321459746?report=genbank&log$=prottop&blast_rank=72&RID=P7CNM1K0015) | *Daphnia pulex* | 62% | 5,00E-80 | 43% |
| [XP_008479791.1](http://www.ncbi.nlm.nih.gov/protein/662212146?report=genbank&log$=prottop&blast_rank=73&RID=P7CNM1K0015) | *Diaphorina citri* | 58% | 3,00E-77 | 47% |
| [KZS06316.1](http://www.ncbi.nlm.nih.gov/protein/1022760053?report=genbank&log$=prottop&blast_rank=74&RID=P7CNM1K0015) | *Daphnia magna* | 63% | 3,00E-77 | 43% |
| [KPJ08867.1](http://www.ncbi.nlm.nih.gov/protein/930667801?report=genbank&log$=prottop&blast_rank=75&RID=P7CNM1K0015) | *Papilio machaon* | 54% | 5,00E-77 | 48% |
| [BAO03859.1](http://www.ncbi.nlm.nih.gov/protein/557356262?report=genbank&log$=prottop&blast_rank=76&RID=P7CNM1K0015) | *Sogatella furcifera* | 62% | 8,00E-75 | 43% |
| [BAO03862.1](http://www.ncbi.nlm.nih.gov/protein/557356268?report=genbank&log$=prottop&blast_rank=77&RID=P7CNM1K0015) | *Laodelphax striatella* | 70% | 2,00E-74 | 40% |
| [ALH22351.1](http://www.ncbi.nlm.nih.gov/protein/936447388?report=genbank&log$=prottop&blast_rank=78&RID=P7CNM1K0015) | *Photuris sp. 1 GJM-2015* | 60% | 4,00E-74 | 40% |
| [BAO03856.1](http://www.ncbi.nlm.nih.gov/protein/557356256?report=genbank&log$=prottop&blast_rank=79&RID=P7CNM1K0015) | *Nilaparvata lugens* | 62% | 6,00E-74 | 42% |
| [ALB48859.1](http://www.ncbi.nlm.nih.gov/protein/924292285?report=genbank&log$=prottop&blast_rank=80&RID=P7CNM1K0015) | *Photuris sp. KSH8870* | 60% | 9,00E-74 | 40% |
| [ALH22349.1](http://www.ncbi.nlm.nih.gov/protein/936447384?report=genbank&log$=prottop&blast_rank=81&RID=P7CNM1K0015) | *Photuris sp. GJM-2015* | 60% | 1,00E-73 | 40% |
| [ALB48858.1](http://www.ncbi.nlm.nih.gov/protein/924292283?report=genbank&log$=prottop&blast_rank=82&RID=P7CNM1K0015) | *Photuris frontalis* | 67% | 1,00E-73 | 38% |
| [ALH22350.1](http://www.ncbi.nlm.nih.gov/protein/936447386?report=genbank&log$=prottop&blast_rank=83&RID=P7CNM1K0015) | *Photuris sp. 2 GJM-2015* | 60% | 1,00E-73 | 40% |
| [XP_011496705.1](http://www.ncbi.nlm.nih.gov/protein/766929243?report=genbank&log$=prottop&blast_rank=84&RID=P7CNM1K0015) | *Ceratosolen solmsi marchali* | 62% | 2,00E-73 | 40% |
| [ALH22342.1](http://www.ncbi.nlm.nih.gov/protein/936447370?report=genbank&log$=prottop&blast_rank=85&RID=P7CNM1K0015) | *Bicellonycha wickershamorum* | 60% | 3,00E-73 | 40% |
| [ALB48841.1](http://www.ncbi.nlm.nih.gov/protein/924292249?report=genbank&log$=prottop&blast_rank=86&RID=P7CNM1K0015) | *Lucidota atra* | 67% | 3,00E-73 | 38% |
| [BAQ54924.1](http://www.ncbi.nlm.nih.gov/protein/761599301?report=genbank&log$=prottop&blast_rank=87&RID=P7CNM1K0015) | *Mnais costalis* | 54% | 4,00E-73 | 47% |

Table S 1

**List of all presumptive Rh7 homologues.** BLAST analysis was performed in <http://blast.ncbi.nlm.nih.gov/Blast.cgi?PAGE=Proteins> using the Rh7 protein sequence.

| Rh7 N-Term | 100% |  |  |  |  |  |  |
| --- | --- | --- | --- | --- | --- | --- | --- |
| Rh1 N-Term | 16% | 100% |  |  |  |  |  |
| Rh2 N-Term | 14.03% | 52% | 100% |  |  |  |  |
| Rh3 N-Term | 17.24% | 22% | 15.78% | 100% |  |  |  |
| Rh4 N-Term | 18.51% | 18% | 20.37% | 53.7% | 100% |  |  |
| Rh5 N-Term | 12% | 8% | 10% | 16% | 22% | 100% |  |
| Rh6 N-Term | 22.22% | 26.66% | 28.88% | 35.55% | 13.33% | 20% | 100% |
|  | Rh7  N-Term | Rh1  N-Term | Rh2  N-Term | Rh3  N-Term | Rh4  N-Term | Rh5  N-Term | Rh6  N-Term |

Table S 2

**Sequence identities between N-termini of all *Drosophila* Rhodopsins in percentage.** Pairwise comparison of amino acid sequences of Rhodopsins (http://imed.med.ucm.es/Tools/sias.html). The N-terminus of all Rhodopsins, with the exception of Rh1/Rh2 and Rh3/Rh4, differ a lot, while the whole protein identities (Table 1) are at least about 30% similar.
