# Supplementary figures and images for: Rhodopsin 7–The unusual Rhodopsin in Drosophila
Source: PeerJ. 2016 Sep 6;4:e2427. doi: 10.7717/peerj.2427 (PMC5018682; doi:10.7717/peerj.2427)

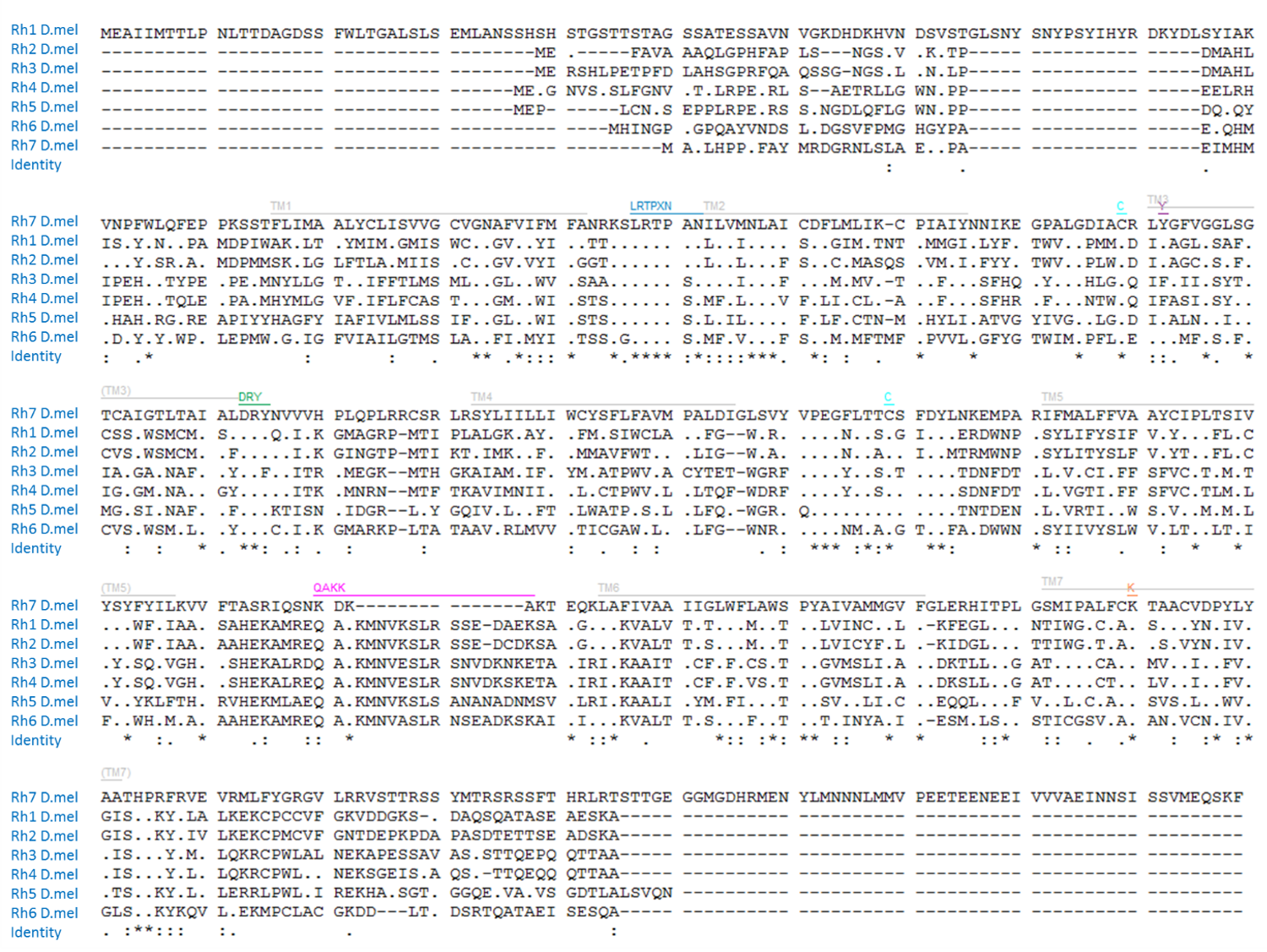

Supplement: Supplemental Information 2 — The transmembrane domains are shown in grey, the LRTPXN motif is shown in blue, the disulfide bridge binding cysteines are shown in cyan, the visual light sensitive tyrosine is shown purple, the DRY motif is shown in green, the chromophore binding lysine is shown in orange, and the proper location of the QAKK motif is shown magenta. [file peerj-04-2427-s002.png]

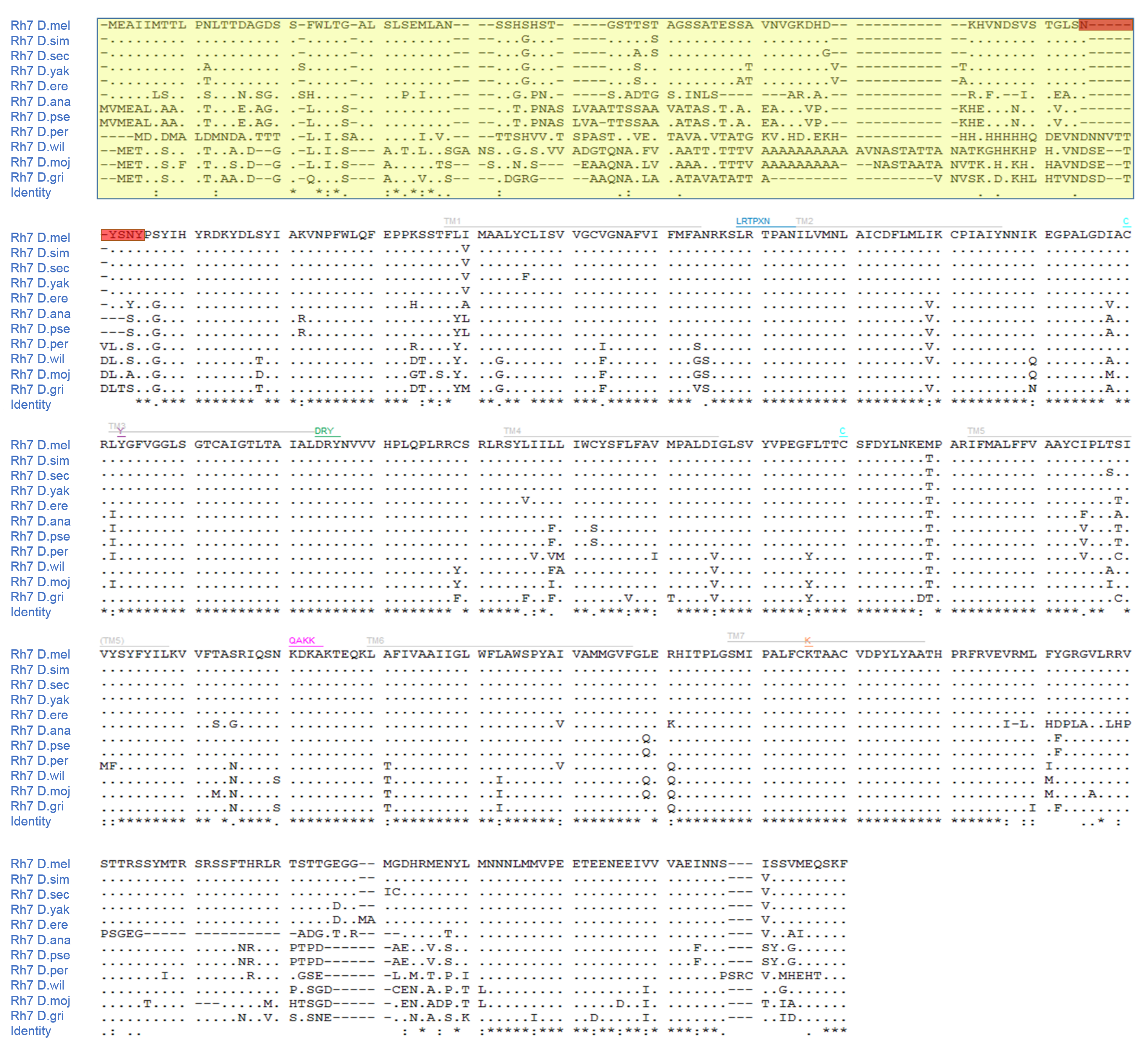

Supplement: Supplemental Information 3 [file peerj-04-2427-s003.png]

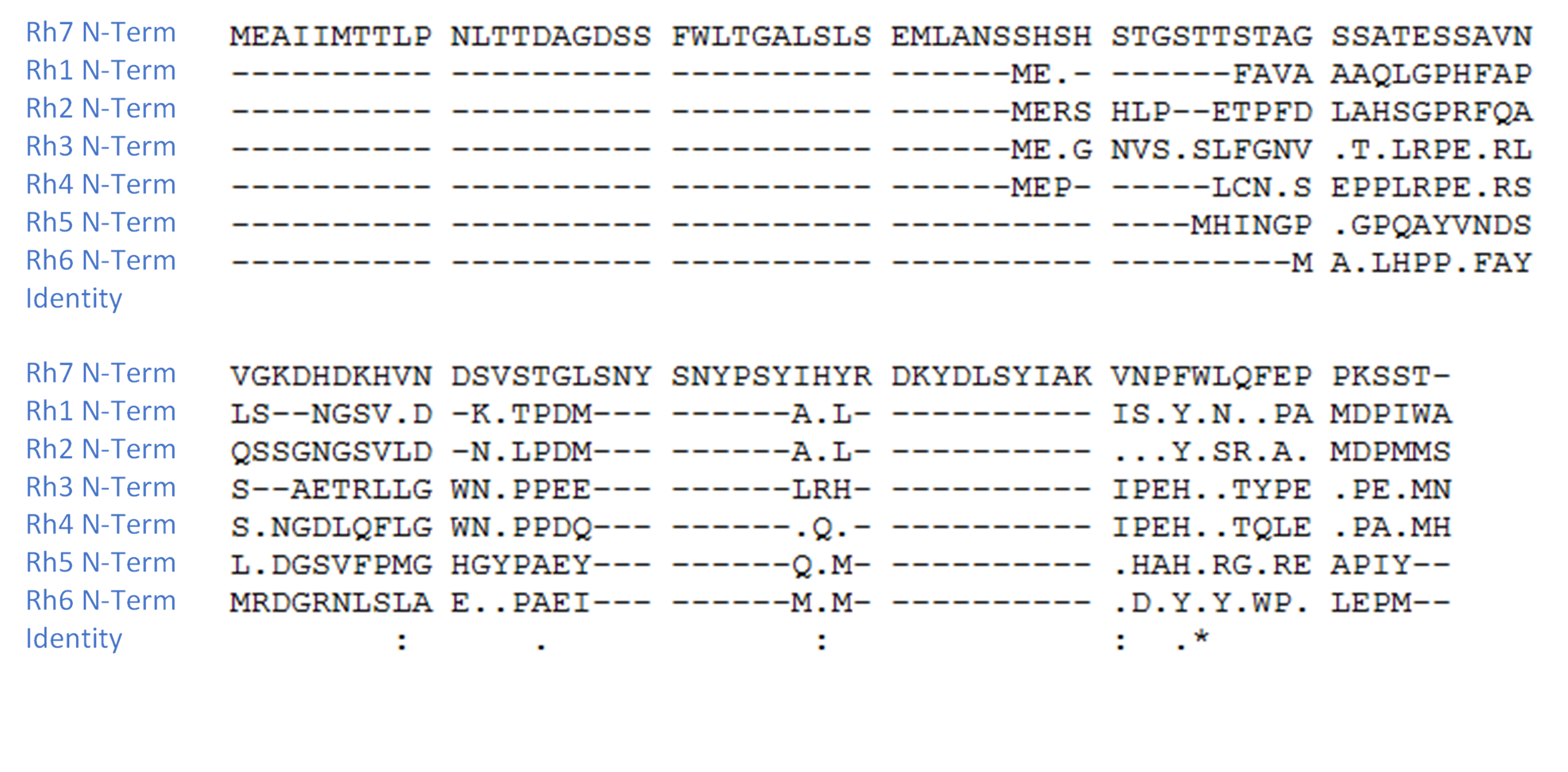

Supplement: Supplemental Information 4 — The N-termini of all Drosophila Rhodopsins were aligned. The amino acid sequences before the first transmembrane domain starts were extracted for the alignment. The N-termini of each Rhodopsin seem to be kept very variable. [file peerj-04-2427-s004.png]

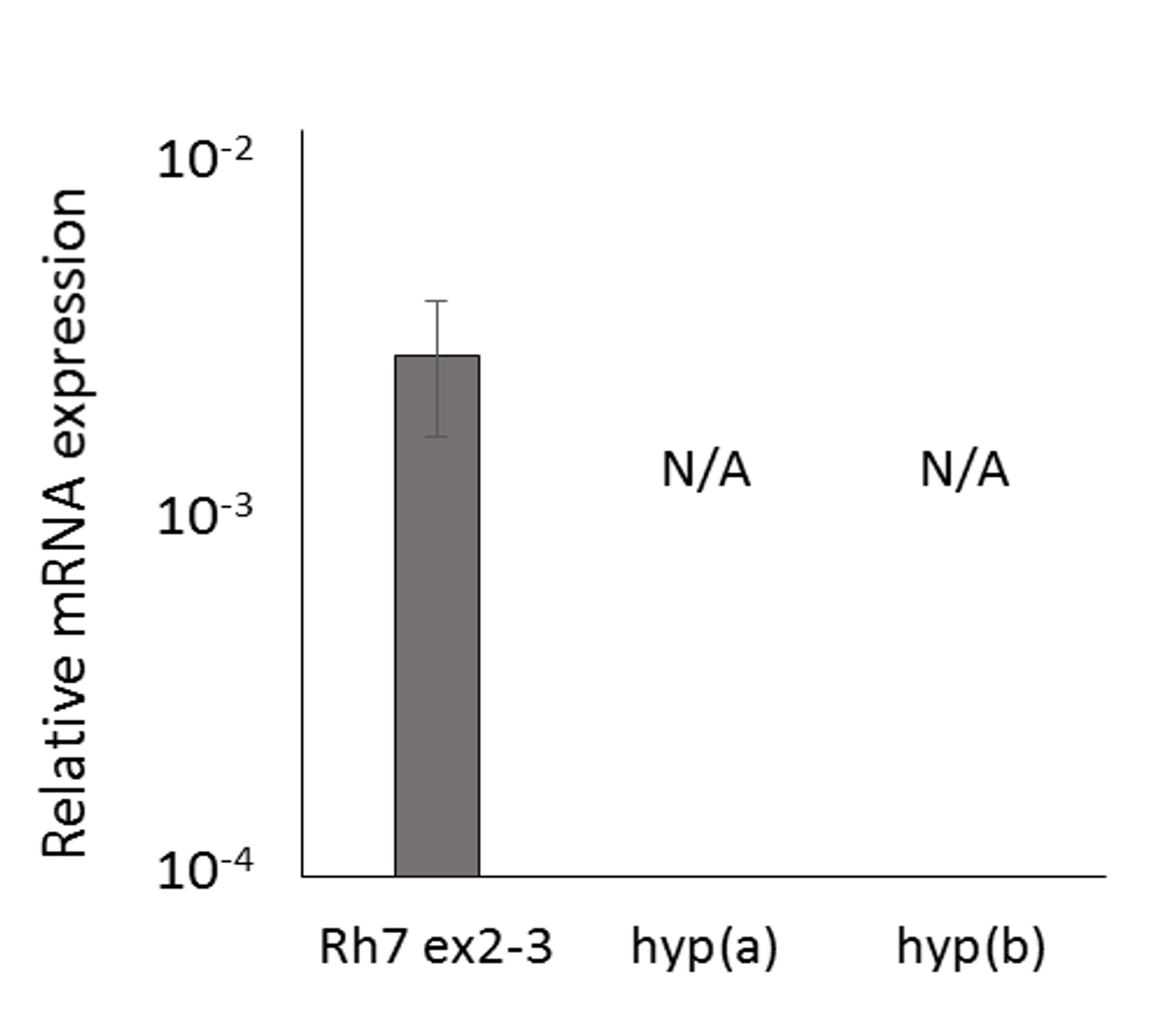

Supplement: Supplemental Information 5 [file peerj-04-2427-s005.png]
