# Supplementary material for: Rhodopsin 7–The unusual Rhodopsin in Drosophila
Source: PeerJ. 2016 Sep 6;4:e2427. doi: 10.7717/peerj.2427 (PMC5018682; doi:10.7717/peerj.2427)
Supplement: Supplemental Information 6 — BLAST analysis was performed in http://blast.ncbi.nlm.nih.gov/Blast.cgi?PAGE=Proteins using the Rh7 protein sequence. [file peerj-04-2427-s006.docx]

| Accession | Organism | Query cover | E value | Ident |
| --- | --- | --- | --- | --- |
| [NP_524035.2](http://www.ncbi.nlm.nih.gov/protein/24663181?report=genbank&log$=prottop&blast_rank=1&RID=P7CNM1K0015) | *Drosophila melanogaster* | 100% | 0% | 100% |
| [XP_002084612.2](http://www.ncbi.nlm.nih.gov/protein/1013917308?report=genbank&log$=prottop&blast_rank=2&RID=P7CNM1K0015) | *Drosophila simulans* | 100% | 0% | 99% |
| [EDX10197.1](http://www.ncbi.nlm.nih.gov/protein/194196621?report=genbank&log$=prottop&blast_rank=3&RID=P7CNM1K0015) | *Drosophila simulans* | 100% | 0% | 99% |
| [XP_002030241.1](http://www.ncbi.nlm.nih.gov/protein/195327063?report=genbank&log$=prottop&blast_rank=4&RID=P7CNM1K0015) | *Drosophila sechellia* | 100% | 0% | 98% |
| [XP_001972505.1](http://www.ncbi.nlm.nih.gov/protein/194869710?report=genbank&log$=prottop&blast_rank=5&RID=P7CNM1K0015) | *Drosophila erecta* | 100% | 0% | 97% |
| [XP_002094554.1](http://www.ncbi.nlm.nih.gov/protein/195493766?report=genbank&log$=prottop&blast_rank=6&RID=P7CNM1K0015) | *Drosophila yakuba* | 100% | 0% | 98% |
| [XP_016934041.1](http://www.ncbi.nlm.nih.gov/protein/1036061592?report=genbank&log$=prottop&blast_rank=7&RID=P7CNM1K0015) | *Drosophila suzukii* | 100% | 0% | 95% |
| [XP_001956024.2](http://www.ncbi.nlm.nih.gov/protein/964135588?report=genbank&log$=prottop&blast_rank=8&RID=P7CNM1K0015) | *Drosophila ananassae* | 100% | 0% | 86% |
| [XP_002134833.1](http://www.ncbi.nlm.nih.gov/protein/198464743?report=genbank&log$=prottop&blast_rank=9&RID=P7CNM1K0015) | *Drosophila pseudoobscura pseudoobscura* | 100% | 0% | 85% |
| [XP_002021096.1](http://www.ncbi.nlm.nih.gov/protein/195160465?report=genbank&log$=prottop&blast_rank=10&RID=P7CNM1K0015) | *Drosophila persimilis* | 100% | 0% | 85% |
| [XP_001983912.1](http://www.ncbi.nlm.nih.gov/protein/195013820?report=genbank&log$=prottop&blast_rank=11&RID=P7CNM1K0015) | *Drosophila grimshawi* | 99% | 0% | 80% |
| [XP_002046729.2](http://www.ncbi.nlm.nih.gov/protein/968115898?report=genbank&log$=prottop&blast_rank=12&RID=P7CNM1K0015) | *Drosophila virilis* | 99% | 0% | 78% |
| [XP_002007363.2](http://www.ncbi.nlm.nih.gov/protein/968041259?report=genbank&log$=prottop&blast_rank=13&RID=P7CNM1K0015) | *Drosophila mojavensis* | 99% | 0% | 81% |
| [XP_002068301.1](http://www.ncbi.nlm.nih.gov/protein/195440958?report=genbank&log$=prottop&blast_rank=14&RID=P7CNM1K0015) | *Drosophila willistoni* | 95% | 0% | 79% |
| [XP_011196234.1](http://www.ncbi.nlm.nih.gov/protein/751443117?report=genbank&log$=prottop&blast_rank=15&RID=P7CNM1K0015) | *Bactrocera cucurbitae* | 83% | 0% | 66% |
| [XP_011201681.1](http://www.ncbi.nlm.nih.gov/protein/751784747?report=genbank&log$=prottop&blast_rank=16&RID=P7CNM1K0015) | *Bactrocera dorsalis* | 83% | 0% | 66% |
| [ALC43468.1](http://www.ncbi.nlm.nih.gov/protein/924558456?report=genbank&log$=prottop&blast_rank=17&RID=P7CNM1K0015) | *Drosophila busckii* | 57% | 2,00E-169 | 88% |
| [XP_014100483.1](http://www.ncbi.nlm.nih.gov/protein/929380214?report=genbank&log$=prottop&blast_rank=18&RID=P7CNM1K0015) | *Bactrocera oleae* | 70% | 4,00E-161 | 68% |
| [KNC24725.1](http://www.ncbi.nlm.nih.gov/protein/906462480?report=genbank&log$=prottop&blast_rank=19&RID=P7CNM1K0015) | *Lucilia cuprina* | 79% | 1,00E-137 | 54% |
| [XP_004525783.1](http://www.ncbi.nlm.nih.gov/protein/498966319?report=genbank&log$=prottop&blast_rank=20&RID=P7CNM1K0015) | *Ceratitis capitata* | 61% | 2,00E-135 | 67% |
| [XP_013114216.1](http://www.ncbi.nlm.nih.gov/protein/907728888?report=genbank&log$=prottop&blast_rank=21&RID=P7CNM1K0015) | *Stomoxys calcitrans* | 73% | 6,00E-126 | 55% |
| [XP_005181535.1](http://www.ncbi.nlm.nih.gov/protein/557763648?report=genbank&log$=prottop&blast_rank=22&RID=P7CNM1K0015) | *Musca domestica* | 84% | 2,00E-125 | 48% |
| [KFB35649.1](http://www.ncbi.nlm.nih.gov/protein/668445239?report=genbank&log$=prottop&blast_rank=23&RID=P7CNM1K0015) | *Anopheles sinensis* | 72% | 2,00E-119 | 51% |
| [XP_308329.4](http://www.ncbi.nlm.nih.gov/protein/158285471?report=genbank&log$=prottop&blast_rank=24&RID=P7CNM1K0015) | *Anopheles gambiae str. PEST* | 67% | 2,00E-118 | 52% |
| [XP_015375898.1](http://www.ncbi.nlm.nih.gov/protein/985387628?report=genbank&log$=prottop&blast_rank=25&RID=P7CNM1K0015) | *Diuraphis noxia* | 69% | 2,00E-113 | 47% |
| [XP_001944926.2](http://www.ncbi.nlm.nih.gov/protein/328719619?report=genbank&log$=prottop&blast_rank=26&RID=P7CNM1K0015) | *Acyrthosiphon pisum* | 68% | 1,00E-109 | 46% |
| [XP_001650744.1](http://www.ncbi.nlm.nih.gov/protein/157109598?report=genbank&log$=prottop&blast_rank=27&RID=P7CNM1K0015) | *Aedes aegypti* | 66% | 5,00E-107 | 49% |
| [XP_001943275.1](http://www.ncbi.nlm.nih.gov/protein/193615573?report=genbank&log$=prottop&blast_rank=28&RID=P7CNM1K0015) | *Acyrthosiphon pisum* | 79% | 6,00E-106 | 42% |
| [XP_012545028.1](http://www.ncbi.nlm.nih.gov/protein/827544844?report=genbank&log$=prottop&blast_rank=29&RID=P7CNM1K0015) | *Bombyx mori* | 65% | 2,00E-105 | 50% |
| [XP_015376154.1](http://www.ncbi.nlm.nih.gov/protein/985421486?report=genbank&log$=prottop&blast_rank=30&RID=P7CNM1K0015) | *Diuraphis noxia* | 71% | 3,00E-105 | 45% |
| [XP_014244894.1](http://www.ncbi.nlm.nih.gov/protein/939250651?report=genbank&log$=prottop&blast_rank=31&RID=P7CNM1K0015) | *Cimex lectularius* | 73% | 6,00E-105 | 46% |
| [BAQ54908.1](http://www.ncbi.nlm.nih.gov/protein/761599219?report=genbank&log$=prottop&blast_rank=32&RID=P7CNM1K0015) | *Ischnura asiatica* | 73% | 8,00E-103 | 47% |
| [XP_013190166.1](http://www.ncbi.nlm.nih.gov/protein/913318290?report=genbank&log$=prottop&blast_rank=33&RID=P7CNM1K0015) | *Amyelois transitella* | 69% | 2,00E-102 | 46% |
| [XP_014366780.1](http://www.ncbi.nlm.nih.gov/protein/943970148?report=genbank&log$=prottop&blast_rank=34&RID=P7CNM1K0015) | *Papilio machaon* | 68% | 4,00E-102 | 47% |
| [XP_001861638.1](http://www.ncbi.nlm.nih.gov/protein/170051159?report=genbank&log$=prottop&blast_rank=35&RID=P7CNM1K0015) | *Culex quinquefasciatus* | 57% | 3,00E-101 | 55% |
| [DAA64804.1](http://www.ncbi.nlm.nih.gov/protein/856116597?report=genbank&log$=prottop&blast_rank=36&RID=P7CNM1K0015) | *Pediculus humanus corporis* | 71% | 7,00E-101 | 44% |
| [XP_014283879.1](http://www.ncbi.nlm.nih.gov/protein/939675733?report=genbank&log$=prottop&blast_rank=37&RID=P7CNM1K0015) | *Halyomorpha halys* | 68% | 9,00E-101 | 47% |
| [KXJ83044.1](http://www.ncbi.nlm.nih.gov/protein/1000213647?report=genbank&log$=prottop&blast_rank=38&RID=P7CNM1K0015) | *Aedes albopictus* | 59% | 6,00E-100 | 51% |
| [XP_013165246.1](http://www.ncbi.nlm.nih.gov/protein/910324350?report=genbank&log$=prottop&blast_rank=39&RID=P7CNM1K0015) | *Papilio xuthus* | 68% | 1,00E-99 | 46% |
| [XP_015511954.1](http://www.ncbi.nlm.nih.gov/protein/998503670?report=genbank&log$=prottop&blast_rank=40&RID=P7CNM1K0015) | *Neodiprion lecontei* | 64% | 2,00E-99 | 49% |
| [XP_015511956.1](http://www.ncbi.nlm.nih.gov/protein/998503672?report=genbank&log$=prottop&blast_rank=41&RID=P7CNM1K0015) | *Neodiprion lecontei* | 64% | 3,00E-99 | 49% |
| [ETN63380.1](http://www.ncbi.nlm.nih.gov/protein/568254415?report=genbank&log$=prottop&blast_rank=42&RID=P7CNM1K0015) | *Anopheles darlingi* | 62% | 3,00E-99 | 51% |
| [XP_015511951.1](http://www.ncbi.nlm.nih.gov/protein/998503664?report=genbank&log$=prottop&blast_rank=43&RID=P7CNM1K0015) | *Neodiprion lecontei* | 61% | 3,00E-99 | 51% |
| [FAA01169.1](http://www.ncbi.nlm.nih.gov/protein/761600095?report=genbank&log$=prottop&blast_rank=44&RID=P7CNM1K0015) | *Ladona fulva* | 71% | 1,00E-98 | 45% |
| [XP_013138793.1](http://www.ncbi.nlm.nih.gov/protein/909566808?report=genbank&log$=prottop&blast_rank=45&RID=P7CNM1K0015) | *Papilio polytes* | 68% | 1,00E-98 | 46% |
| [BAQ54717.1](http://www.ncbi.nlm.nih.gov/protein/761598532?report=genbank&log$=prottop&blast_rank=46&RID=P7CNM1K0015) | *Orthetrum albistylum* | 63% | 2,00E-98 | 48% |
| [BAQ54849.1](http://www.ncbi.nlm.nih.gov/protein/761598865?report=genbank&log$=prottop&blast_rank=47&RID=P7CNM1K0015) | *Anax parthenope* | 86% | 5,00E-98 | 39% |
| [BAQ54884.1](http://www.ncbi.nlm.nih.gov/protein/761599033?report=genbank&log$=prottop&blast_rank=48&RID=P7CNM1K0015) | *Epiophlebia superstes* | 63% | 8,00E-98 | 48% |
| [XP_011549645.1](http://www.ncbi.nlm.nih.gov/protein/768417961?report=genbank&log$=prottop&blast_rank=49&RID=P7CNM1K0015) | *Plutella xylostella* | 74% | 8,00E-98 | 43% |
| [BAQ54939.1](http://www.ncbi.nlm.nih.gov/protein/761599391?report=genbank&log$=prottop&blast_rank=50&RID=P7CNM1K0015) | *Indolestes peregrinus* | 79% | 2,00E-97 | 42% |
| [XP_012266578.1](http://www.ncbi.nlm.nih.gov/protein/817087588?report=genbank&log$=prottop&blast_rank=51&RID=P7CNM1K0015) | *Athalia rosae* | 62% | 3,00E-97 | 50% |
| [BAQ54814.1](http://www.ncbi.nlm.nih.gov/protein/761598795?report=genbank&log$=prottop&blast_rank=52&RID=P7CNM1K0015) | *Tanypteryx pryeri* | 80% | 1,00E-96 | 42% |
| [BAQ54741.1](http://www.ncbi.nlm.nih.gov/protein/761598652?report=genbank&log$=prottop&blast_rank=53&RID=P7CNM1K0015) | *Somatochlora uchidai* | 65% | 8,00E-96 | 48% |
| [BAQ54697.1](http://www.ncbi.nlm.nih.gov/protein/761598310?report=genbank&log$=prottop&blast_rank=54&RID=P7CNM1K0015) | *Sympetrum frequens* | 76% | 1,00E-95 | 43% |
| [BAQ54792.1](http://www.ncbi.nlm.nih.gov/protein/761598753?report=genbank&log$=prottop&blast_rank=55&RID=P7CNM1K0015) | *Anotogaster sieboldii* | 63% | 2,00E-95 | 48% |
| [BAQ54766.1](http://www.ncbi.nlm.nih.gov/protein/761598701?report=genbank&log$=prottop&blast_rank=56&RID=P7CNM1K0015) | *Macromia amphigena* | 65% | 9,00E-94 | 47% |
| [XP_013165248.1](http://www.ncbi.nlm.nih.gov/protein/910324354?report=genbank&log$=prottop&blast_rank=57&RID=P7CNM1K0015) | *Papilio xuthus* | 63% | 2,00E-92 | 47% |
| [BAQ54832.1](http://www.ncbi.nlm.nih.gov/protein/761598831?report=genbank&log$=prottop&blast_rank=58&RID=P7CNM1K0015) | *Asiagomphus melaenops* | 61% | 4,00E-92 | 47% |
| [XP_013794217.1](http://www.ncbi.nlm.nih.gov/protein/926606621?report=genbank&log$=prottop&blast_rank=59&RID=P7CNM1K0015) | *Limulus polyphemus* | 69% | 2,00E-91 | 42% |
| [ANF89423.1](http://www.ncbi.nlm.nih.gov/protein/1031182501?report=genbank&log$=prottop&blast_rank=60&RID=P7CNM1K0015) | *Limulus polyphemus* | 69% | 3,00E-91 | 42% |
| [XP_002432663.1](http://www.ncbi.nlm.nih.gov/protein/242024495?report=genbank&log$=prottop&blast_rank=61&RID=P7CNM1K0015) | *Pediculus humanus corporis* | 63% | 4,00E-91 | 45% |
| [XP_001687896.1](http://www.ncbi.nlm.nih.gov/protein/158285473?report=genbank&log$=prottop&blast_rank=62&RID=P7CNM1K0015) | *Anopheles gambiae str. PEST* | 52% | 3,00E-89 | 53% |
| [ANF89422.1](http://www.ncbi.nlm.nih.gov/protein/1031182499?report=genbank&log$=prottop&blast_rank=63&RID=P7CNM1K0015) | *Limulus polyphemus* | 72% | 1,00E-88 | 41% |
| [XP_013773163.1](http://www.ncbi.nlm.nih.gov/protein/926616116?report=genbank&log$=prottop&blast_rank=64&RID=P7CNM1K0015) | *Limulus polyphemus* | 68% | 2,00E-88 | 42% |
| [KPI91300.1](http://www.ncbi.nlm.nih.gov/protein/930648952?report=genbank&log$=prottop&blast_rank=65&RID=P7CNM1K0015) | *Papilio xuthus* | 59% | 5,00E-88 | 47% |
| [KOB66307.1](http://www.ncbi.nlm.nih.gov/protein/914557755?report=genbank&log$=prottop&blast_rank=66&RID=P7CNM1K0015) | *Operophtera brumata* | 69% | 9,00E-87 | 41% |
| [XP_013165247.1](http://www.ncbi.nlm.nih.gov/protein/910324352?report=genbank&log$=prottop&blast_rank=67&RID=P7CNM1K0015) | *Papilio xuthus* | 58% | 9,00E-87 | 48% |
| [XP_013138794.1](http://www.ncbi.nlm.nih.gov/protein/909566810?report=genbank&log$=prottop&blast_rank=68&RID=P7CNM1K0015) | *Papilio polytes* | 59% | 3,00E-86 | 47% |
| [XP_015791070.1](http://www.ncbi.nlm.nih.gov/protein/1005966130?report=genbank&log$=prottop&blast_rank=69&RID=P7CNM1K0015) | *Tetranychus urticae* | 62% | 1,00E-85 | 45% |
| [XP_015784405.1](http://www.ncbi.nlm.nih.gov/protein/1005952087?report=genbank&log$=prottop&blast_rank=70&RID=P7CNM1K0015) | *Tetranychus urticae* | 65% | 5,00E-83 | 41% |
| [EFX70801.1](http://www.ncbi.nlm.nih.gov/protein/321459751?report=genbank&log$=prottop&blast_rank=71&RID=P7CNM1K0015) | *Daphnia pulex* | 64% | 6,00E-81 | 42% |
| [EFX70796.1](http://www.ncbi.nlm.nih.gov/protein/321459746?report=genbank&log$=prottop&blast_rank=72&RID=P7CNM1K0015) | *Daphnia pulex* | 62% | 5,00E-80 | 43% |
| [XP_008479791.1](http://www.ncbi.nlm.nih.gov/protein/662212146?report=genbank&log$=prottop&blast_rank=73&RID=P7CNM1K0015) | *Diaphorina citri* | 58% | 3,00E-77 | 47% |
| [KZS06316.1](http://www.ncbi.nlm.nih.gov/protein/1022760053?report=genbank&log$=prottop&blast_rank=74&RID=P7CNM1K0015) | *Daphnia magna* | 63% | 3,00E-77 | 43% |
| [KPJ08867.1](http://www.ncbi.nlm.nih.gov/protein/930667801?report=genbank&log$=prottop&blast_rank=75&RID=P7CNM1K0015) | *Papilio machaon* | 54% | 5,00E-77 | 48% |
| [BAO03859.1](http://www.ncbi.nlm.nih.gov/protein/557356262?report=genbank&log$=prottop&blast_rank=76&RID=P7CNM1K0015) | *Sogatella furcifera* | 62% | 8,00E-75 | 43% |
| [BAO03862.1](http://www.ncbi.nlm.nih.gov/protein/557356268?report=genbank&log$=prottop&blast_rank=77&RID=P7CNM1K0015) | *Laodelphax striatella* | 70% | 2,00E-74 | 40% |
| [ALH22351.1](http://www.ncbi.nlm.nih.gov/protein/936447388?report=genbank&log$=prottop&blast_rank=78&RID=P7CNM1K0015) | *Photuris sp. 1 GJM-2015* | 60% | 4,00E-74 | 40% |
| [BAO03856.1](http://www.ncbi.nlm.nih.gov/protein/557356256?report=genbank&log$=prottop&blast_rank=79&RID=P7CNM1K0015) | *Nilaparvata lugens* | 62% | 6,00E-74 | 42% |
| [ALB48859.1](http://www.ncbi.nlm.nih.gov/protein/924292285?report=genbank&log$=prottop&blast_rank=80&RID=P7CNM1K0015) | *Photuris sp. KSH8870* | 60% | 9,00E-74 | 40% |
| [ALH22349.1](http://www.ncbi.nlm.nih.gov/protein/936447384?report=genbank&log$=prottop&blast_rank=81&RID=P7CNM1K0015) | *Photuris sp. GJM-2015* | 60% | 1,00E-73 | 40% |
| [ALB48858.1](http://www.ncbi.nlm.nih.gov/protein/924292283?report=genbank&log$=prottop&blast_rank=82&RID=P7CNM1K0015) | *Photuris frontalis* | 67% | 1,00E-73 | 38% |
| [ALH22350.1](http://www.ncbi.nlm.nih.gov/protein/936447386?report=genbank&log$=prottop&blast_rank=83&RID=P7CNM1K0015) | *Photuris sp. 2 GJM-2015* | 60% | 1,00E-73 | 40% |
| [XP_011496705.1](http://www.ncbi.nlm.nih.gov/protein/766929243?report=genbank&log$=prottop&blast_rank=84&RID=P7CNM1K0015) | *Ceratosolen solmsi marchali* | 62% | 2,00E-73 | 40% |
| [ALH22342.1](http://www.ncbi.nlm.nih.gov/protein/936447370?report=genbank&log$=prottop&blast_rank=85&RID=P7CNM1K0015) | *Bicellonycha wickershamorum* | 60% | 3,00E-73 | 40% |
| [ALB48841.1](http://www.ncbi.nlm.nih.gov/protein/924292249?report=genbank&log$=prottop&blast_rank=86&RID=P7CNM1K0015) | *Lucidota atra* | 67% | 3,00E-73 | 38% |
| [BAQ54924.1](http://www.ncbi.nlm.nih.gov/protein/761599301?report=genbank&log$=prottop&blast_rank=87&RID=P7CNM1K0015) | *Mnais costalis* | 54% | 4,00E-73 | 47% |
