# Supplementary material for: Rhodopsin 7–The unusual Rhodopsin in Drosophila
Source: PeerJ. 2016 Sep 6;4:e2427. doi: 10.7717/peerj.2427 (PMC5018682; doi:10.7717/peerj.2427)
Supplement: Supplemental Information 7 — Pairwise comparison of amino acid sequences of Rhodopsins (http://imed.med.ucm.es/Tools/sias.html). The N-terminus of all Rhodopsins, with the exception of Rh1/Rh2 and Rh3/Rh4, differ a lot, while the whole protein identities (Table 1) are at least about 30% similar. [file peerj-04-2427-s007.docx]

| Rh7 N-Term | 100% |  |  |  |  |  |  |
| --- | --- | --- | --- | --- | --- | --- | --- |
| Rh1 N-Term | 16% | 100% |  |  |  |  |  |
| Rh2 N-Term | 14.03% | 52% | 100% |  |  |  |  |
| Rh3 N-Term | 17.24% | 22% | 15.78% | 100% |  |  |  |
| Rh4 N-Term | 18.51% | 18% | 20.37% | 53.7% | 100% |  |  |
| Rh5 N-Term | 12% | 8% | 10% | 16% | 22% | 100% |  |
| Rh6 N-Term | 22.22% | 26.66% | 28.88% | 35.55% | 13.33% | 20% | 100% |
|  | Rh7  N-Term | Rh1  N-Term | Rh2  N-Term | Rh3  N-Term | Rh4  N-Term | Rh5  N-Term | Rh6  N-Term |
